# Analysis and numerical calculation of a coupled creep and strain-softening model for soft rock tunnels

**DOI:** 10.1371/journal.pone.0256243

**Published:** 2021-08-26

**Authors:** Jianjun Zhang, Baicong Yao, Yunhe Ao, Chunzhe Jin, Chuang Sun

**Affiliations:** School of Civil Engineering, Liaoning Technical University, Fuxin, Liaoning, China; University of Vigo, SPAIN

## Abstract

Proper mechanical model selection is critical in tunnel support design and stability analysis, especially to reflect the creep and strain-softening behavior of soft rock. We present a coupled nonlinear Burgers strain-softening (NBSS) model and numerical calculation method to investigate the coupled effects of creep and strain-softening of soft rock tunnels. The nonlinear elastic-viscous model is used to simulate the steady creep behavior of mudstone, and the nonlinear viscoplastic strain-softening model is used to simulate the accelerated creep behavior and post-peak strength attenuation behavior. The experimental results show that the viscoplastic parameters and post-peak softening parameters of mudstone are highly sensitive to confining pressure and exhibit nonlinear characteristics. The accelerated creep curve obtained by the numerical calculation is consistent with the experiments, which verifies the model reliability. We use the NBSS and nonlinear Burgers Mohr-Coulomb (NBMC) models to calculate the plastic zone distribution characteristics and deformation law. The distribution of the plastic zone calculated by the NBSS model is larger with more localized fractures. The NBSS model is useful for studying the evolution of stress and displacement fields of complex surrounding rock mass, which provides important theoretical guidelines for the support design and stability analysis of soft rock tunnel engineering.

## Introduction

Rock strength attenuation and creep characteristics control the stability of supporting structures during tunnel excavation and support engineering [1–5]. The material strength characteristics of surrounding rock strength gradually decreases with time, particularly in soft rock settings [6,7]. Soft rock creep prior to reaching the peak value typically undergoes decay and steady creep. Under loading, the micro-cracks in soft rock expand over time and the rock strength gradually decreases with increasing viscoplastic strain. When the strength is reduced to the load stress level, the soft rock will enter the accelerated creep stage. The strength attenuation process of soft rock over time can be characterized by creep damage and strain-softening models [8–11].

Previous rock creep damage studies focused mainly on how to determine creep damage variables and applied mechanical models to describe accelerated rock creep. Because the rheological process of surrounding rock is very complex in practical engineering and creep parameters tend to change with the stress environment, some previous studies developed an unsteady fractional order creep model to investigate the characteristics of accelerated rock creep [12–16]. The unsteady fractional order creep model well reflects the creep acceleration stage but is impractical for model development and calculations and still requires basic elastic-viscoplastic theory as a support [17–19]. Gioda and Sterpi [20] considered that rock viscoplastic strain is the root cause of rheological instability in tunnel engineering. Other scholars began from rock elasto-viscoplasticity theory and constructed rock creep damage models. For example, Zhao et al. [21] proposed an elasto-viscoplastic rheological creep damage model and data processing algorithm using experiments and data processing methods of rock visco-elastic and visco-plastic strain separation, and quantified the creep model parameters by linear interpolation [22]. Fu et al. [23] built a three-dimensional discrete element grain-based stress corrosion model incorporating the theories of subcritical crack growth and chemical reaction rate to explore the time-dependent behavior of damage evolution and fracture patterns of brittle rocks on a mesoscopic scale. Manica et al. [24] proposed a rheological constitutive model of mudstone developed within an elastic-plastic framework, which completely describes the rheological characteristics of mudstone. Zhang et al. [25] proposed a novel classifier ensemble to improve prediction accuracy for tunnel squeezing problems by aggregating seven individual classifiers using the weighted voting method.

Within the study of post-peak rock strength attenuation characteristics, previous studies have mainly constructed different types of rock strain-softening models. For example, Alejano et al. [26] constructed a mechanical model of post-peak rock strength attenuation based on elastoplastic mechanics theory using a geological strength index of the surrounding rock rating system [27]. Krajcinovic and Silva [28] established a statistical constitutive model of rock strain-softening damage using a random distribution of internal defects and rock microelement strength following a Weibull distribution [29]. Sun et al. [30] determined the relationship between post-peak softening modulus and confining pressure of granite using nonlinear fitting methods and constructed a strain-softening mechanical model of granite according to the geometric relationship of stress-strain space. Feng et al. [31] developed a strain-softening damage model of the rocks with defect growth based on damage evolution. Wang et al. [32] proposed a new method for the numerical calculation of rock strain-softening processes.

Abundant achievements have been made in the study of damage creep and strength attenuation of rock. However, an approach to study the coupled law of creep and strain-softening in soft rock remains poorly defined, as well as how to numerically reflect the accelerated creep characteristics of soft rock and strength attenuation law after accelerated creep.

To address this issue, we analyzed the coupled mechanism of creep and strain-softening of soft rock surrounding a tunnel using theoretical methods and performed laboratory experiments to study the strain-softening and creep damage characteristics of mudstone. We construct a nonlinear Burgers creep damage model and Mohr-Coulomb strain-softening model based on the nonlinear exponential fitting method, and propose numerical calculation methods using a nonlinear Burgers Mohr-Coulomb (NBMC) model and nonlinear Burgers strain-softening (NBSS) model. We calculate the coupled effect law of creep and strain-softening of rock surrounding a circular tunnel using numerical simulations, and analyze the distribution characteristics of the plastic zone and deformation evolution law of surrounding rock.

## Theoretical bases

### Strain-softening behavior of rock mass

**Fig 1:**
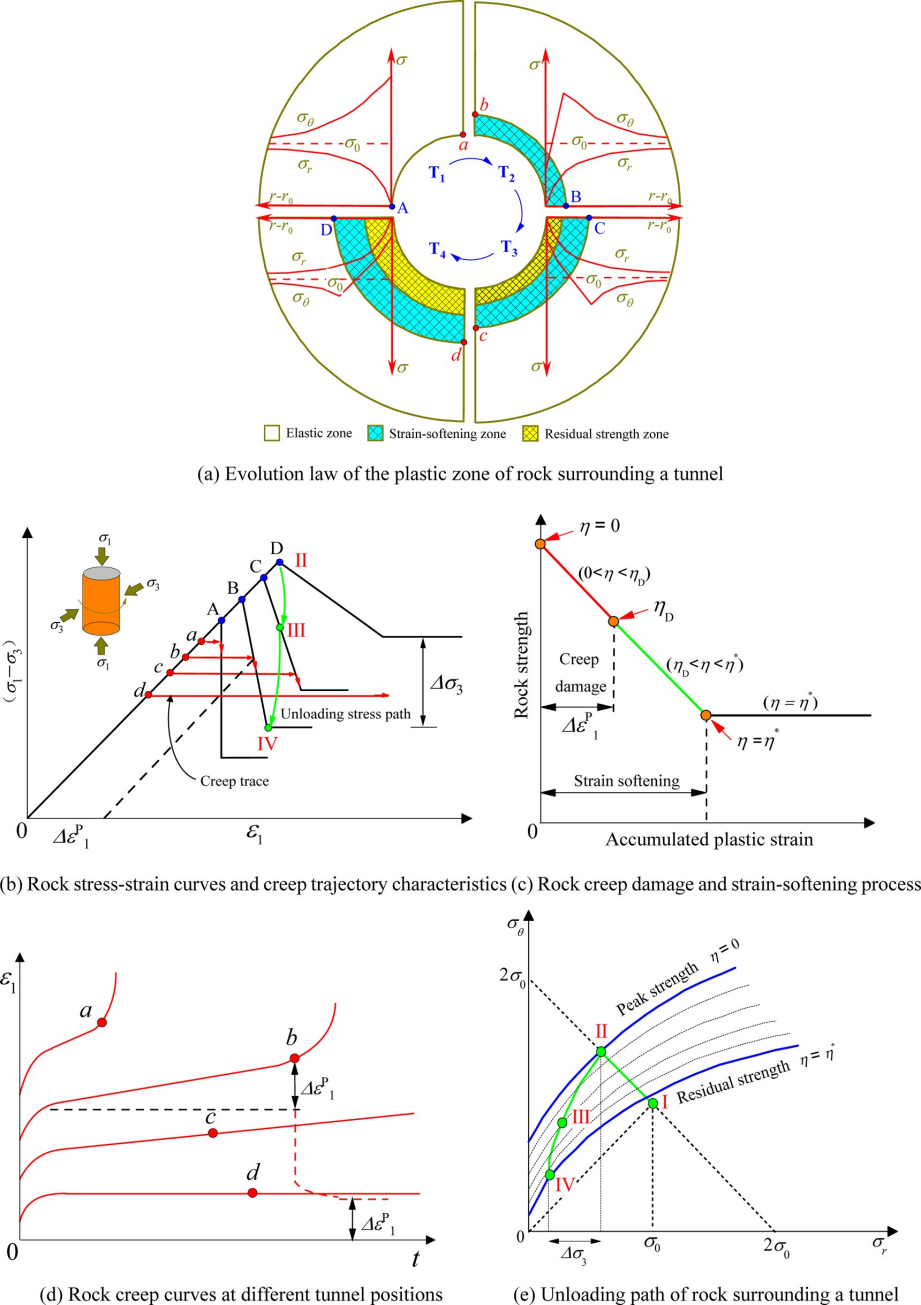
Coupled relationship between creep and strain-softening of rock surrounding a tunnel. (a) Evolution law of the plastic zone of rock surrounding a tunnel (b) Rock stress-strain curves and creep trajectory characteristics (c) Rock creep damage and strain-softening process (d) Rock creep curves at different tunnel positions (e) Unloading path of rock surrounding a tunnel.

A hypothetical two-dimensional circular soft rock tunnel is shown in Fig 1A. The surrounding rock is in a state of hydrostatic pressure, the original rock stress is *σ*_0_, and the excavation radius is *r*_0_. Without considering rock creep, the tangential stress *σ*_*θ*_ at point A approaches 2*σ*_0_ and the radial stress *σ*_*r*_ approaches 0 owing to the unloading of the surrounding rock at time T_1_. At this time, the surrounding rock at point A is within a state similar to uniaxial compression. If *σ*_*θ*_ − *σ*_*r*_ is greater than the surrounding rock strength at point A, a plastic strain-softening zone gradually forms in the surrounding rock. At T_2_, if *σ*_*θ*_ − *σ*_*r*_ is greater than the rock strength at point B, the post-peak rock strength will attenuate with increasing cumulative plastic strain. By analogy, after T_3_ and T_4_, the plastic zone tends to stabilize after points C and D reach the yield strength and the surrounding rock between points A and B reaches the residual strength. At this time, the surrounding rock of the tunnel is composed of three parts: an elastic zone; a plastic strain-softening zone; and a plastic residual zone. The stress-strain curves of the surrounding rock at points A, B, C, and D can be approximately expressed as a simplified three-line segment, as shown in Fig 1B.

The process by which the strength of rock surrounding a tunnel gradually attenuates with post-peak cumulative plastic strain can be characterized by the plastic softening coefficient *η*, which has a nonlinear relationship with the plastic strain. It is assumed that the stress component expression of the rock mass yield condition considering damage is as follows:

$$F(\sigma_{ij,}\;\eta (t))=0$$ (1)

When the surrounding rock reaches the peak strength, its strength parameters gradually change with increasing *η*. The *η* value is assumed to decrease to η* and the rock strength reaches the residual strength. This relationship can be expressed as [27]:

$$\omega (\eta )=\{\begin{array}{l} \omega^{p}-\frac{\omega^{p}-\omega^{r}}{\eta^{*}}\eta ,\;0<\eta <\eta^{*}\\ \omega^{r},\;\eta \geq \eta^{*} \end{array}$$ (2)

where *ω* is the rock strength parameter, *ω*^*p*^ is the peak strength parameter, and *ω*^*r*^ is the residual strength parameter. The variation law is shown in Fig 1C. The process by which *η* decreases from 0 to η* is called surrounding rock strain-softening.

### Coupled behavior of rock creep and strain-softening

Under rock creep conditions, the unloading of rock surrounding a tunnel concentrate stress concentration at point *a* at T_1_, as shown in Fig 1A. The concentrated stress *σ*_*θ*_ at point *a* is considered to be less than the peak strength of the surrounding rock. The creep damage deformation of the surrounding rock at point *a* increases with time owing to the rock creep damage characteristics. The creep trajectory of the surrounding rock at point *a* gradually extends to the post-peak stress-strain curve of point A. Accelerated creep failure occurs when the strength of the surrounding rock is less than *σ*_*θ*_ owing to creep damage. The creep trajectory is shown in Fig 1B and the creep curve of the corresponding surrounding rock is shown in Fig 1D. After creep failure of the surrounding rock at point *a*, its strength will continue to attenuate. According to the stress-strain curve characteristics at point A, the post-peak strength of the surrounding rock continues to attenuate to the residual strength with increasing cumulative plastic strain. At T_2_, the surrounding rock at point *b* is affected by *σ*_*θ*_ − *σ*_*r*_ and its creep trajectory gradually extends to the post-peak stress-strain curve of point B. After accelerated creep failure, the strength of the surrounding rock at point *b* continues to decrease to the residual strength with increasing cumulative plastic shear strain. After T_3_ and T_4_, the *σ*_*θ*_ − *σ*_*r*_ stress level of the surrounding rock at point *d* is relatively small. The relationship between the creep trajectory and post-peak stress-strain curve at point D is shown in Fig 1B. Creep deformation of the surrounding rock tends to be stable at this stress level, and creep damage does not accelerate creep failure of the surrounding rock at point *d*. The creep curve is shown in Fig 1D. At this time, the plastic zone of the surrounding rock is essentially stable.

The creep damage variable *D* is used to characterize the attenuation process of surrounding rock strength with time and is related to the cumulative plastic strain. With increasing cumulative rock viscoplastic strain, damage cracks appear and the strength gradually attenuates. Accelerated creep failure occurs when the cumulative rock plastic strain at point *b* reaches *Δε*_1_. The creep damage variable *D* is assumed to be *η*_D_, as shown in Fig 1C. After accelerated rock creep damage, the surrounding rock strength continues to decrease with increasing cumulative plastic strain until reaching the residual strength. As the rock creep damage changes to accelerated creep, *η* increases from 0 to *η*_D_. After accelerated creep, *η* increases from *η*_D_ to η^*^ while approaching the residual strength. The transition from pre-peak creep damage to post-peak residual strength is called rock strain-softening.

### Nonlinear behavior of rock failure

When discussing the creep damage and strain-softening process in Fig 1A, the basic assumption is that the *σ*_*r*_ of each point in the surrounding rock remains constant. While the confining pressure of the surrounding rock remains constant, the *σ*_*r*_ of the surrounding rock in the unloading path of the surrounding rock in the same area changes constantly. The unloading path of point D is shown in Fig 1E. When *η* = 0, the surrounding rock at point D is in the pre-peak state; when *η* = η^*^, the surrounding rock at point D is in the residual strength state. After reaching the peak strength, the stress at point D changes from II to III and ultimately reaches the residual strength IV. The variation range of the confining pressure can be expressed by Δ*σ*_3_, as shown in Fig 1B.

In practical engineering, the creep damage and strain-softening of tunnel soft rock show complex nonlinear characteristics. A nonlinear mechanical model should therefore be established in accordance with the creep damage and strength attenuation characteristics of soft rock to better analyze its deformation and failure characteristics in tunnel settings and ultimately propose reasonable control methods.

## Materials and methods

### Rock specimen

Specimens were collected from the surrounding rock face of the DK77+684 section of the Milin tunnel on the Sichuan-Tibet railway. The content of clay minerals is more than 50%, the content of carbonate minerals is less than 25%, and the content of felsic minerals is less than 25%. The yellow mudstone is relatively dense, with an average porosity of 3.29% and water content of 15.7%. The surrounding rock was mainly yellow mudstone with distinct rheological properties. To study the deformation mechanism, we performed conventional triaxial compression tests to determine the rock strain-softening mechanical properties and triaxial creep tests to investigate the rock creep characteristics.

### Triaxial compression tests

#### Experimental protocol and results

**Fig 2:**
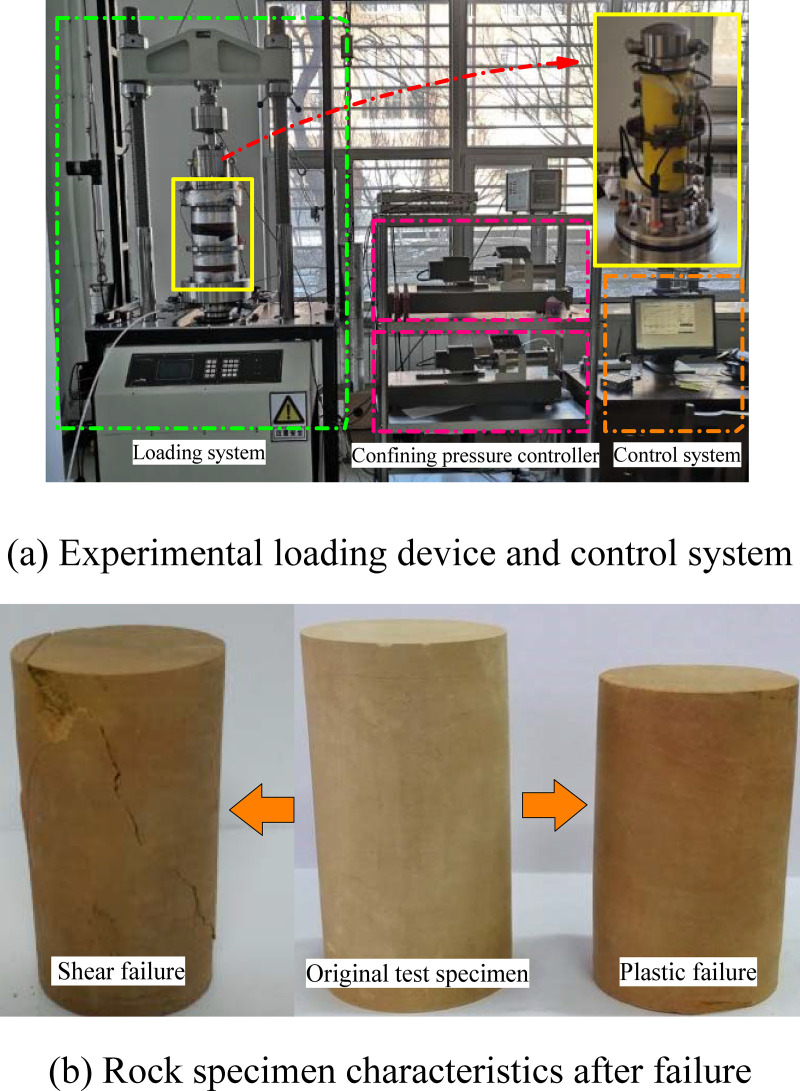
Experimental system and rock specimen characteristics after failure. (a) Experimental loading device and control system (b) Rock specimen characteristics after failure.

A British GDS high precision soft rock rheometer was used in the test, which included a computer and full servo motor control that automatically collected data. The system has good dynamic response function, including a 250-kN motor driven digital load rack, 32-MPa pressure/volume control system, local strain sensor, and multi-function test module. The rheometer has high testing accuracy and meets the requirements of conventional triaxial and creep tests. The experimental loading device and control system are shown in Fig 2A, and the characteristics of the rock specimens after loading failure are shown in Fig 2B.

**Fig 3:**
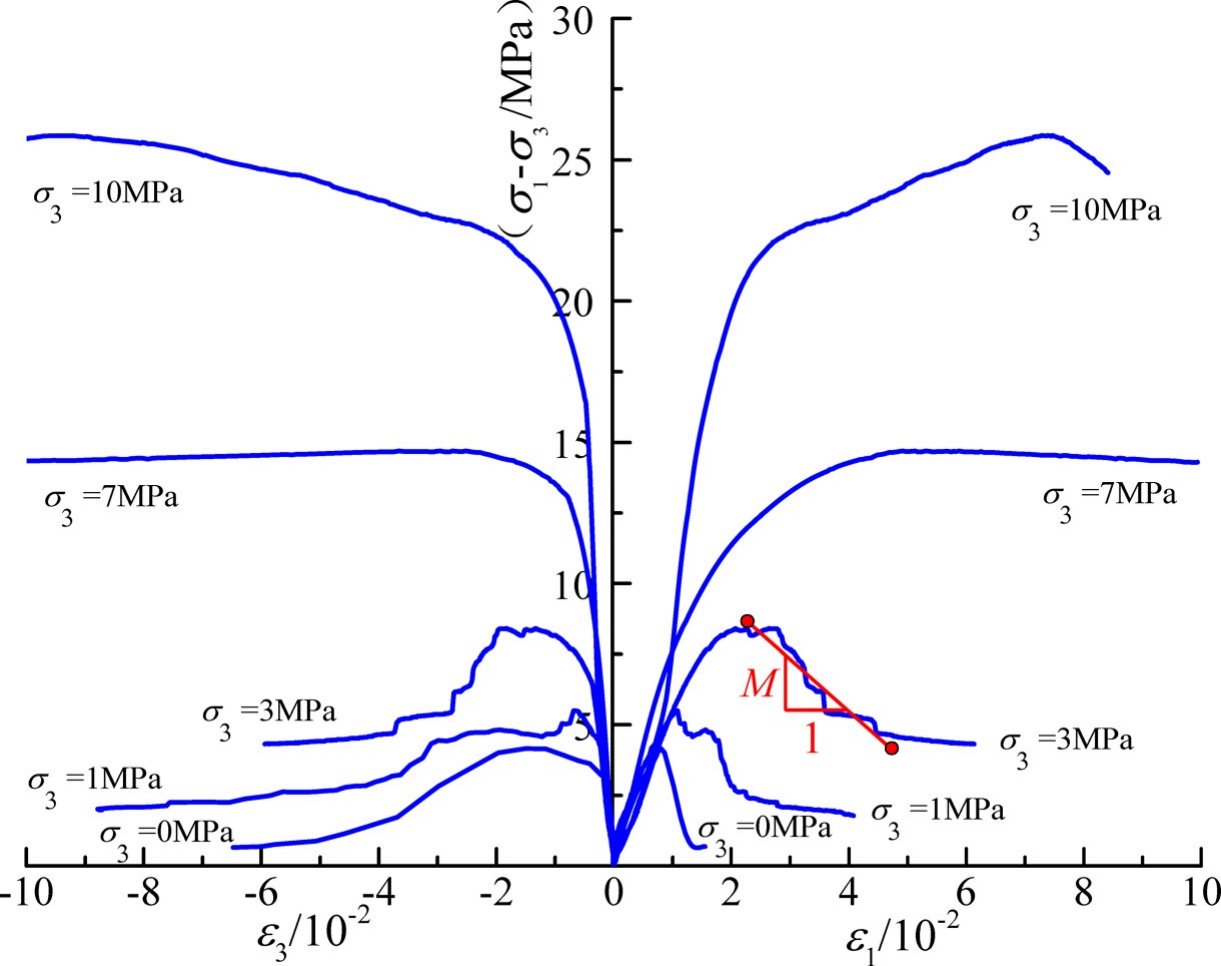
Full stress-strain curves of the mudstone.

The displacement control method was used in the triaxial compression tests and the confining pressures were set to 0, 1, 3, 7, and 10 MPa. The full stress-strain curves of the specimens under different confining pressures were obtained by applying an axial load at the loading rate of 0.1 MPa/min, as shown in Fig 3. The elastic modulus of the mudstone shows good consistency under different confining pressures, varying between 0.86 and 0.92 GPa. Under uniaxial conditions, the specimens exhibited brittle failure characteristics and the post-peak strength approached zero. With increasing confining pressure, the stress-strain curve gradually flatted after the peak value. Under a confining pressure of 7 MPa, the samples show an ideal elastic-plastic state; at 10 MPa, the samples show strain hardening characteristics. The triaxial compression test results show a strong correlation between mudstone strength and confining pressure. The samples show clear brittle-ductile characteristics after reaching the peak value.

#### Peak strength and residual strength parameters

**Fig 4:**
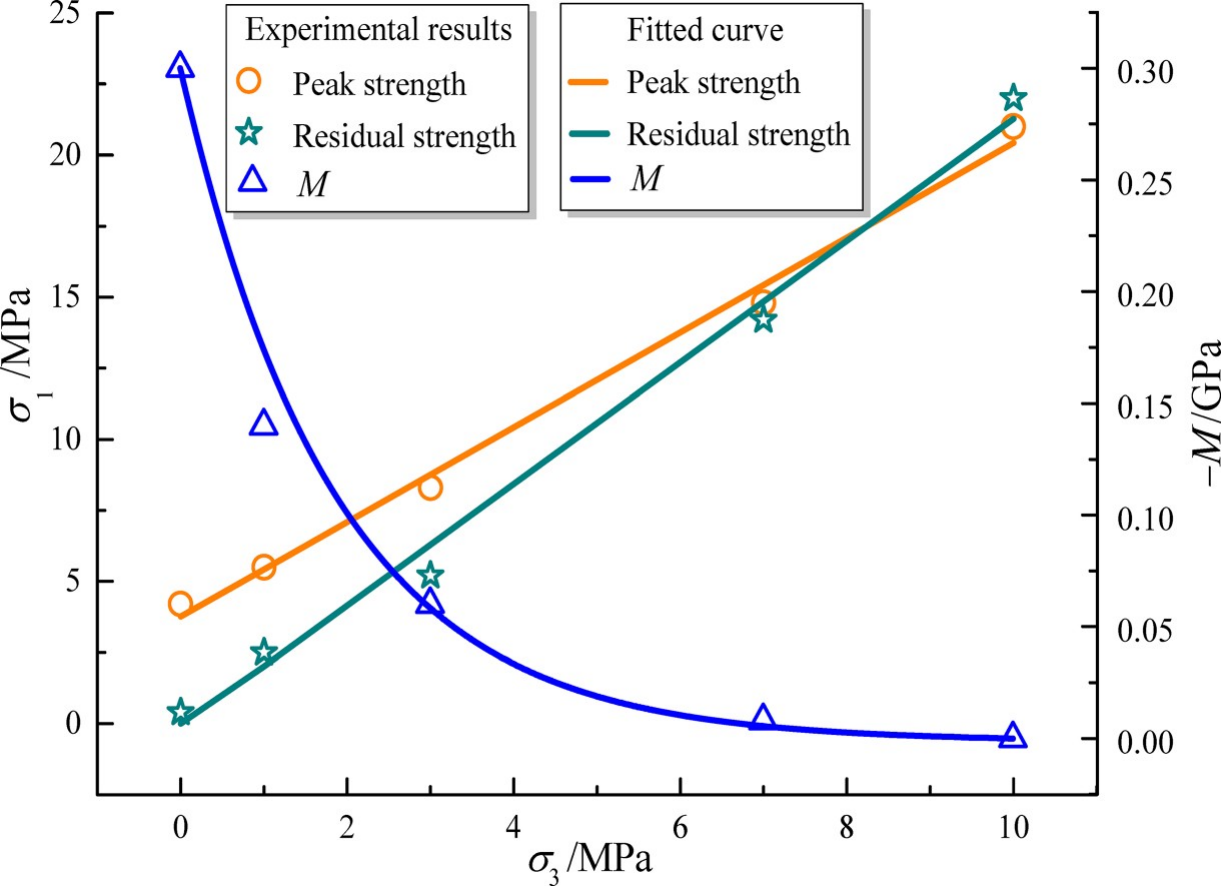
Fitted curves of mudstone strength and softening modulus.

The mudstone strength curve based on the Mohr-Coulomb criterion is obtained by linear fitting the stress combination of peak strength and residual strength of mudstone [1], as shown in Fig 4. The variance values, *R*^2^, of the fitted parameters are all above 0.95, which indicates high fitting accuracy. The peak and residual compressive strength, shear strength and tensile strength parameters based on Mohr-Coulomb criterion were calculated, as shown in Table 1.

**Table 1:** Mudstone strength parameters.

Strength criterion	Mohr-Coulomb criterion				
Strength parameters	*σ*_c_/MPa	*c*/MPa	*Φ*/^o^	*R* ^2^	*σ*_t_/MPa
Peak	4.6	1.62	16.5	0.993	0.36
Residual	0.5	0.45	18.2	0.991	

We use *M* to represent the post-peak stress-strain of the mudstone, i.e. post-peak softening modulus, as shown in Fig 4. The experimental analysis shows a nonlinear correlation between the post-peak softening modulus and confining pressure, which is obtained by nonlinear exponential fitting. The fitted equation is as follows:

$$Y=ae^{\left(-\sigma_{3}/b\right)}+c$$ (3)

where *Y* is the target parameter, which represents softening modulus *M* in this equation, *σ*_3_ is confining pressure, and *a*, *b*, and *c* are the constants obtained by the fitting (Table 2).

**Table 2:** Fitted softening modulus of mudstone.

Target parameter	Fitted parameters			
*Y*	*a*	*b*	*c*	*R* ^2^
*M*	0.289	1.454	0.00681	0.985

### Creep tests

#### Experimental protocol

Mudstone creep tests were carried out using a British GDS high precision soft rock rheometer. We adopted hierarchical loading and applied a maximum load of 80%–90% of the peak strength from the conventional triaxial compression tests under the same confining pressure. The tests were divided into 6–8 loading gradients, as shown in Table 3. During the creep tests, the confining pressure was loaded to the target value using a stress loading rate is 0.05 MPa/s and then held fixed. The axial compression was loaded to the target value of the first stage creep and held constant. The following load was applied every 48 h until the specimen failed.

**Table 3:** Stress loading gradients under different confining pressure.

Confining pressure *σ*_3_/MPa	0	1.0	3.0	7.0
Deviator stress (*σ*_1_-*σ*_3_)/MPa	0.5	0.5	1.0	2.0

#### Creep curve characteristics and fitted parameters

**Fig 5:**
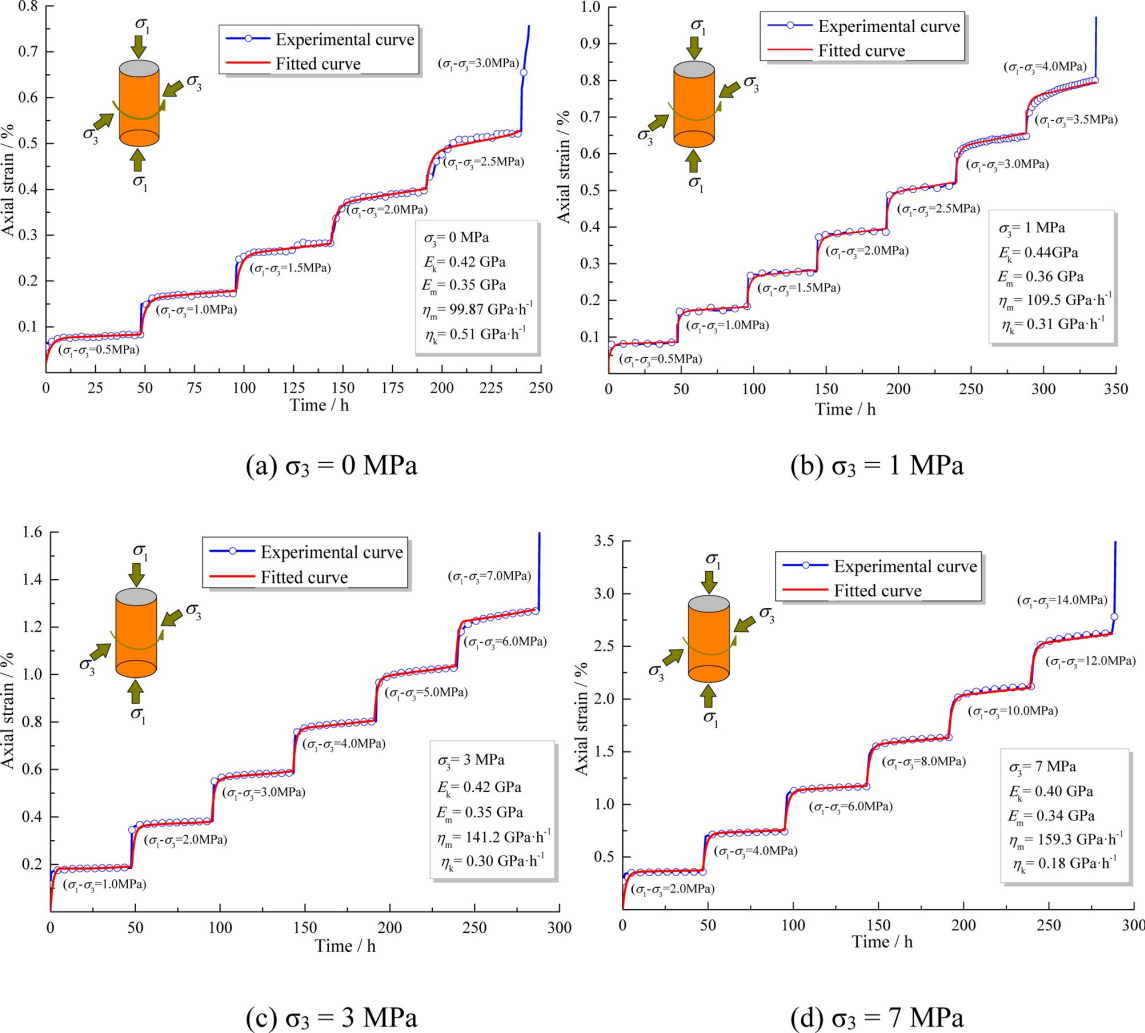
Creep curves and fitted curves of mudstone under different confining pressures. (a) σ_3_ = 0 MPa (b) σ_3_ = 1 MPa (c) σ_3_ = 3 MPa(d) σ_3_ = 7 MPa.

Fig 5 shows the axial creep curves of the mudstone under different confining pressures. The creep curve exhibits the typical three-stage creep characteristics of rock. When *σ*_3_ = 0, the mudstone shows characteristics of decay creep and steady creep while loading from stages 1 to 5. When the deviatoric stress reached 3 MPa, the mudstone entered the accelerated creep stage and the rock specimen was destroyed. The mudstone creep curves characteristics under different confining pressures show that the steady creep curve under high deviatoric stress gradually tends to flatten with the increasing confining pressure. This shows that confining pressure exerts a strong influence on the viscoplastic rheological properties of mudstone.

The Burgers model was used to fit and analyze the creep experimental curve of the mudstone. The Burgers model is composed of a Maxwell model and Kelvin model in series, which well reflects the decay creep and steady creep stage of rock. The specific methods are as follows: The GDS file obtained from the experiment was imported into an Excel file and the experimental data were processed by the Boltzmann superposition principle. The expression is as follows [33]:

$$\varepsilon_{T}(t)=\sum \varepsilon_{i}(t)=\sum \Delta \sigma_{i}J(t-t_{i})\;t>t_{i}$$ (4)

where *J* is the creep compliance, *t* is the creep time, and *i* is the number of hierarchical loading stages, which are taken as 1, 2,…, *i* − 1.

**Fig 6:**
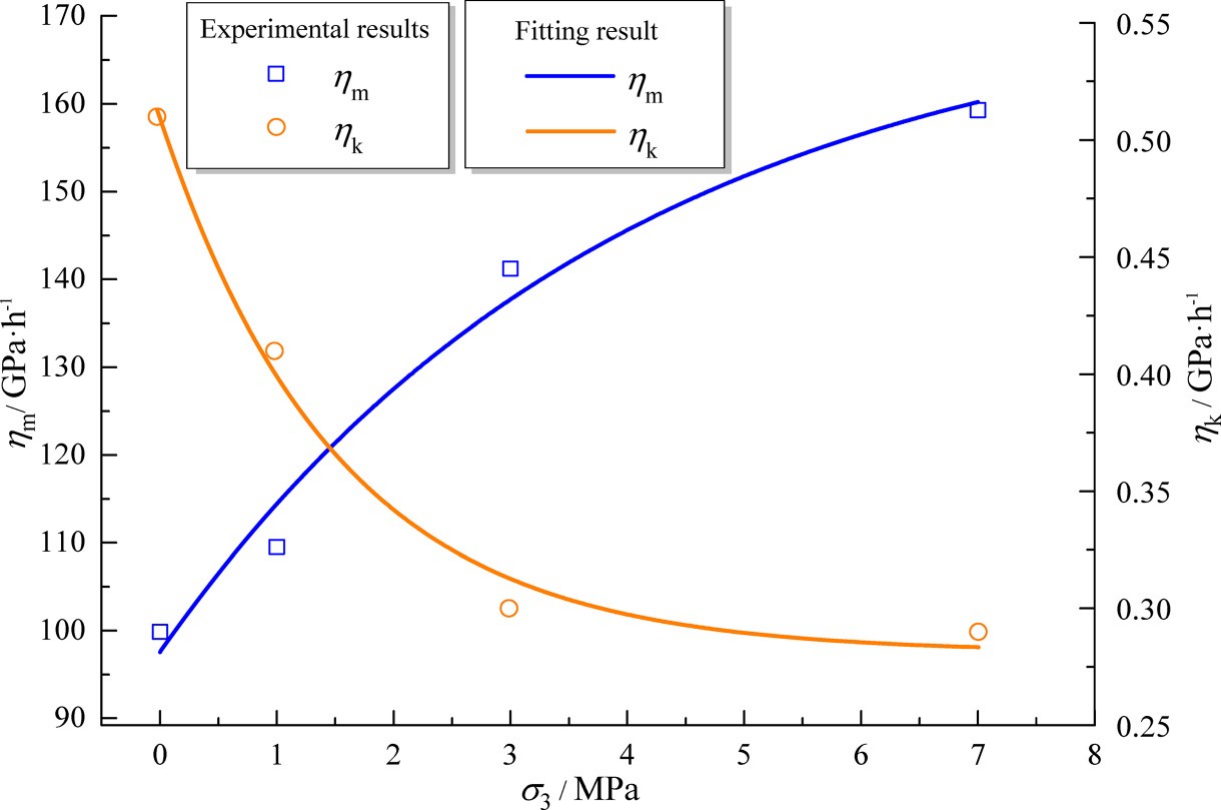
Viscosity coefficient of mudstone as a function of confining pressure.

The processed data were then imported into Origin software. Because the tests were performed in stages, the data were fitted step by step. The obtained nonlinear fitted equation of the Burgers model suitable for the hierarchical loading mode is given as:

$$\begin{array}{l} y=At+\sum \left[A(t-48(i-1))\right]+2B+\\ C\{2-\exp(-Dt)-\sum \left[-D(t-48(i-1))\right]\} \end{array}$$ (5)

where *t* is creep time, *y* is creep strain, *A* = *σ*/*η*_m_, *B* = *σ*/*E*_m_, *C* = *σ*/*E*_k_, and *D* = *E*_k_/*η*_k_. The model parameters *η*_m_, *η*_k_, *E*_m_, and *E*_k_ were inversely calculated according to the fitted results. The obtained parameters are listed in Table 4 and the fitted and experimental curves are compared in Fig 6.

**Table 4:** Fitted creep parameters of mudstone.

*σ*_3_/MPa	*η*_m_/GPa·h^-1^	*η*_k_/GPa·h^-1^	*E*_m_/GPa	*E*_k_/GPa
0	99.87	0.51	0.35	0.42
1	109.5	0.41	0.36	0.44
3	141.2	0.30	0.35	0.42
7	159.3	0.29	0.34	0.40

The effect of confining pressure on the viscosity coefficients based on the Maxwell model and Kelvin model is shown in Fig 6. The viscosity coefficient *η*_*k*_ decreases strongly with increasing confining pressure in the low pressure range (0–3 MPa) and tends to stabilize in the higher pressure range (3–7 MPa). The viscosity coefficient *η*_*m*_ increases gradually with the increase of confining pressure. The relationship between the viscosity coefficient and confining pressure is obtained by exponential fitting analysis of the viscosity coefficients *η*_*m*_ and *η*_*k*_ from the Maxwell and Kelvin models, respectively. The data are fitted following Eq (3) and the obtained parameters are listed in Table 5. The *R*^2^ of the fitted equations all exceed 0.95, which indicates high accuracy.

**Table 5:** Fitted parameters of viscosity coefficient of mudstone.

Target parameters	Fitted parameters			
*Y*	*a*	*b*	*c*	*R* ^2^
*η*_k_	0.23	1.51	0.28	0.97
*η*_m_	-75.54	3.95	173.07	0.95

## NBSS model and numerical implementation

Irreversible viscoplastic deformation occurs during the steady mudstone creep that leads to gradual strength attenuation. Accelerated creep failure occurs when the strength is less than or equal to the load stress level [34,35]. In practical engineering, mudstone strength continues to decrease to the residual strength after creep failure.

### Strain-softening model

In the plastic strain-softening model of rock, the Mohr-Coulomb yield criterion can be expressed as follows [36]:

$$f(\sigma_{\theta },\sigma_{r},\eta )=\sigma_{\theta }-N_{\varphi }(\eta )\sigma_{r}-\sigma_{c}$$ (6)

where *σ*_*c*_ is the uniaxial compressive strength of the rock and:

$$\begin{array}{l} \sigma_{c}=2c(\eta )\sqrt{K_{\varphi }(\eta )}\\ N_{\varphi }=\frac{1+\sin\varphi }{1-\sin\varphi } \end{array}\}$$ (7)

If *c* and *φ* in the Mohr-Coulomb constant are assumed to vary with *η*, the Mohr-Coulomb strain-softening relation in Eq (2) can be obtained and *c* and *φ* can be replaced by *ω*. Strain-softening in mudstone is controlled by the softening modulus *M*. The critical value that mudstones reaches in the residual strength stage, η*, can be expressed in the form of internal variables. The plastic parameter η* can be defined as the plastic shear strain, which is obtained by the difference between the maximum and minimum principal plastic strains as follows [27]:

$$\eta^{*}=\varepsilon_{1}^{p}-\varepsilon_{3}^{p}$$ (8)

where $\varepsilon_{1}^{p}$ and $\varepsilon_{3}^{p}$ are the maximum and minimum plastic strains, respectively.

**Fig 7:**
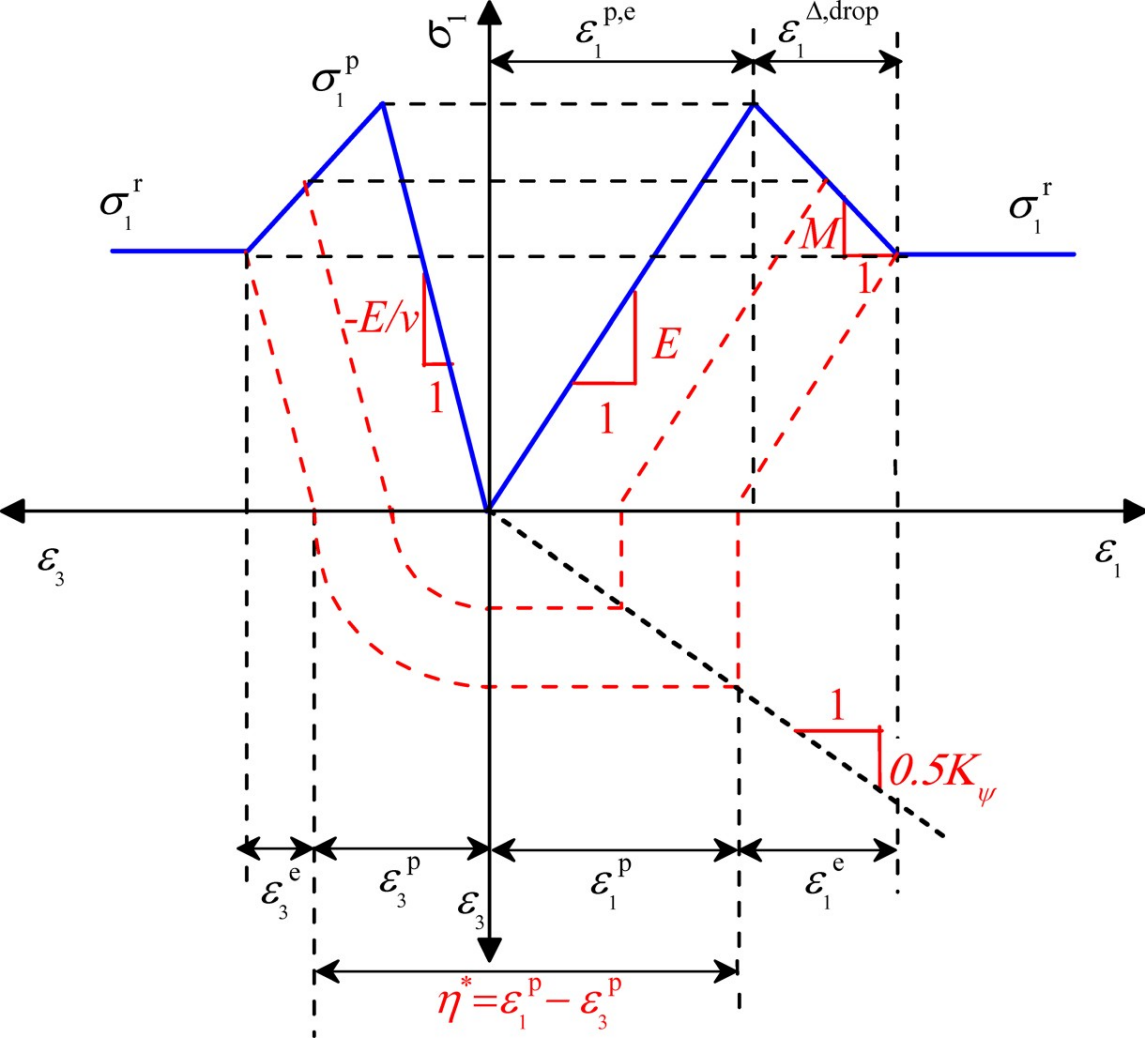
Simplified stress-strain curve of mudstone.

According to the geometric relationship of $\varepsilon_{1}^{p}$ and $\varepsilon_{3}^{p}$ in Fig 7, we obtain:

$$\varepsilon_{1}^{p}=\varepsilon_{1}^{p,e}+\varepsilon_{1}^{\mathrm{drop}}-\varepsilon_{1}^{e}$$ (9)

where $\varepsilon_{1}^{p,e}$ is the maximum elastic principal strain prior to reaching the peak value, $\varepsilon_{1}^{drop}$ is the softening strain after reaching the peak value, and $\varepsilon_{1}^{e}$ is the maximum elastic principal strain. When *σ*_3_ is constant, the parameters in Eq (9) can be expressed as follows:

$$\begin{array}{l} \varepsilon_{1}^{p,e}=\frac{\sigma_{1}^{p}(\sigma_{3})}{E}\\ \varepsilon_{1}^{\mathrm{drop}}=\frac{\sigma_{1}^{p}(\sigma_{3})-\sigma_{1}^{r}(\sigma_{3})}{-M}\\ \varepsilon_{1}^{e}=\frac{\sigma_{1}^{r}(\sigma_{3})}{E} \end{array}\}$$ (10)

where $\sigma_{1}^{p}$ is the peak principal stress and $\sigma_{1}^{r}$ is the residual principal stress.

Considering the dilatancy angle *ψ* and form of the strain increment, we obtain [37]:

$$\sin\psi =\frac{\varepsilon {\&}_{1}^{p}+2\varepsilon {\&}_{3}^{p}}{-\varepsilon {\&}_{1}^{p}+2\varepsilon {\&}_{3}^{p}}$$ (11)

where ${\varepsilon \&}_{j}^{p}$ (*j* = 1, 3) is the plastic principal strain rate.

Eq (11) can be transformed into:

$$\varepsilon {\&}_{3}^{p}=-\frac{1}{2}N_{\psi }\cdot \varepsilon {\&}_{1}^{p}$$ (12)

where:

$$N_{\psi }=\frac{1+\sin\psi }{1-\sin\psi }$$ (13)

Under static conditions, the relationship between $\varepsilon_{3}^{p}$ and $\varepsilon_{1}^{p}$ can be constructed by holding *ψ* fixed according to Eq (12) as follows:

$$\varepsilon_{3}^{p}=-\frac{1}{2}N_{\psi }\cdot \varepsilon_{1}^{p}$$ (14)

The nonlinear expression of η* is then simultaneously obtained by Eqs (8)–(14) as follows:

$$\eta^{*}=\frac{\sigma_{1}^{p}(\sigma_{3})‐\sigma_{1}^{r}(\sigma_{3})}{E}\frac{\xi +1}{\xi }(1+\frac{N_{\psi }}{2})$$ (15)

where:

ξ=−M/E (16)

## NBSS creep damage model

**Fig 8:**
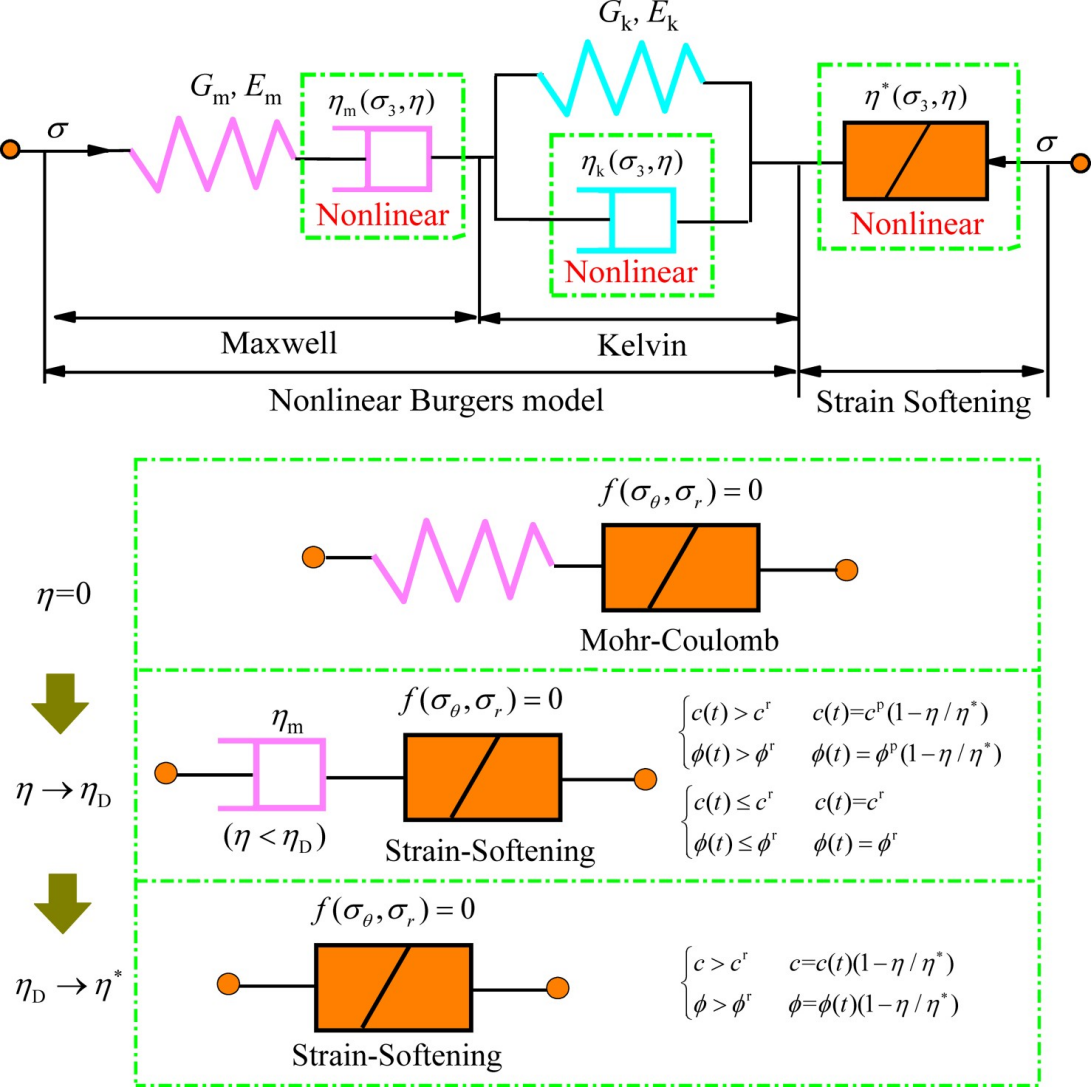
NBSS creep damage model of mudstone.

In this paper, we introduce a nonlinear element based on the Mohr-Coulomb strain-softening and Burgers model. The values of *η*_m_ and *η*_k_ in the Burgers model vary nonlinearly with *σ*_3_ of the surrounding rock. The nonlinear Burgers model is connected in series with the nonlinear strain-softening element to build the NBSS creep damage model, as shown in Fig 8. When *η* = 0, the model is in an elastic-plastic state and whether the rock satisfies the Mohr-Coulomb yield condition is tested under conditions of stress *σ*. When *η* < *η*_D_, the plastic parameter *η*_D_ is taken as the creep damage variable threshold and the model is in the elastic-viscoplastic creep damage state. The strength parameters *c* and *φ* change with time *t* and *η*, and the peak strength parameters *c*^p^ and *ϕ*^p^ transform into residual strength parameters *c*^r^ and *ϕ*^r^. The model enters the accelerated creep state when *η* = *η*_D_ and the plastic strain-softening state when *η*_D_ < *η* < η*. At this time, *c* and *φ* continue to change with *η* until reaching *c*^r^ and *ϕ*^r^ when the rock reaches the residual strength.

The Burgers creep model can reasonably describe the decay creep and steady creep processes, and the stress-strain deviator constitutive relation is [38]:

$$2E_{m}\dot{e_{ij}}+2\eta_{m}{\overset{¨}{e}}_{ij}=\frac{E_{m}}{\eta_{k}}S_{ij}+(1+\frac{E_{m}}{E_{k}}+\frac{\eta_{m}}{\eta_{k}}){\dot{S}}_{ij}+\frac{\eta_{m}}{E_{k}}{\overset{¨}{S}}_{ij}$$ (17)

The axial rock creep strain under deviator stress (-) and confining pressure *σ*_3_ can be deduced according to the Burgers model as:

$$\varepsilon (t)=\frac{\sigma_{1}-\sigma_{3}}{9K}+\frac{\sigma_{1}-\sigma_{3}}{3G_{m}}+\frac{\sigma_{1}-\sigma_{3}}{3G_{k}}[1-\exp(-\frac{G_{m}t}{\eta_{m}})]+\frac{\sigma_{1}-\sigma_{3}}{3\eta_{k}}t$$ (18)

where:

$$\begin{array}{l} G_{m}=\frac{E_{m}}{2(1+\nu )}\\ G_{k}=\frac{E_{k}}{2(1+\nu )} \end{array}\}$$ (19)

Based on the Mohr-Coulomb criterion, *f* = 0, the shear yield and tensile yield are obtained in the principal axis stress space as follows [36]:

$$f_{s}=\sigma_{1}-\sigma_{3}N_{\varphi }+2c\sqrt{N_{\varphi }}$$ (20)

$$f=\sigma_{t}-\sigma_{3}$$ (21)

where *σ*_*t*_ is the tensile strength.

Let *c*^p^ and *φ*^p^ be the initial cohesion and internal friction angle of rock, respectively. During the creep process, *c* and *φ* of rock vary with the plastic parameter as:

$$c(t)=c^{p}(1-\eta /\eta^{*})$$ (22)

$$\varphi (t)=\varphi^{p}(1-\eta /\eta^{*})$$ (23)

where *η* varies with *η*_m_. By substituting Eqs (22) and (23) into Eqs (20) and (21), the time-dependent shear yield criterion of the nonlinear strain-softening plastic elements can be obtained:

$$f_{s}=\sigma_{1}-\sigma_{3}N_{\varphi }+2c(t)\sqrt{N_{\varphi }}$$ (24)

$$N_{\varphi }=\frac{1+\sin\varphi (t)}{1-\sin\varphi (t)}$$ (25)

In FLAC^3D^, for the Kelvin model:

$$S_{ij}=2\eta_{m}{\dot{e}}_{{}_{ij}}^{K}+2E_{m}e_{ij}^{K}$$ (26)

and for the Maxwell model:

$${\dot{e}}_{{}_{ij}}^{M}=\frac{{\dot{S}}_{ij}}{2E_{k}}+\frac{{\overset{}{S}}_{ij}}{2\eta_{k}}$$ (27)

The deviator strain rate of nonlinear strain-softening model is:

$${\dot{e}}_{{}_{ij}}^{P}=\lambda \frac{\partial g}{\partial \sigma_{ij}}-\frac{1}{3}{\dot{e}}_{vol}^{P}\delta_{ij}$$ (28)

$${\dot{e}}_{{}_{vol}}^{P}=\lambda [\frac{\partial g}{\partial \sigma_{11}}+\frac{\partial g}{\partial \sigma_{22}}+\frac{\partial g}{\partial \sigma_{33}}]$$ (29)

The volume behavior is given as:

$${\dot{\sigma }}_{0}=K({\dot{e}}_{vol}-{\dot{e}}_{vol}^{P})$$ (30)

In FLAC^3D^, the plastic parameter *η* is expressed by the plastic shear strain, and its increment form is given as:

$$\Delta \varepsilon^{\mathrm{ps}}=\frac{\sqrt{2}}{2}\sqrt{{\left(\Delta \varepsilon_{{}^{1}}^{\mathrm{ps}}‐\Delta \varepsilon_{m}^{\mathrm{ps}}\right)}^{2}+{\left(\Delta \varepsilon_{m}^{\mathrm{ps}}\right)}^{2}+{\left(\Delta \varepsilon_{3}^{\mathrm{ps}}‐\Delta \varepsilon_{m}^{\mathrm{ps}}\right)}^{2}}$$ (31)

where:

$$\Delta \varepsilon_{m}^{\mathrm{ps}}=(\Delta \varepsilon_{1}^{\mathrm{ps}}+\Delta \varepsilon_{3}^{\mathrm{ps}})/2$$ (32)

where $\Delta \varepsilon_{j}^{ps}$ (*j* = 1, 3) is the main plastic shear strain increment. When considering rock dilatancy behavior, the unrelated flow law corresponding to the plastic shear potential function can be expressed as:

$$g^{s}=\sigma_{1}-\sigma_{3}N_{\psi }$$ (33)

### Model establishment

**Fig 9:**
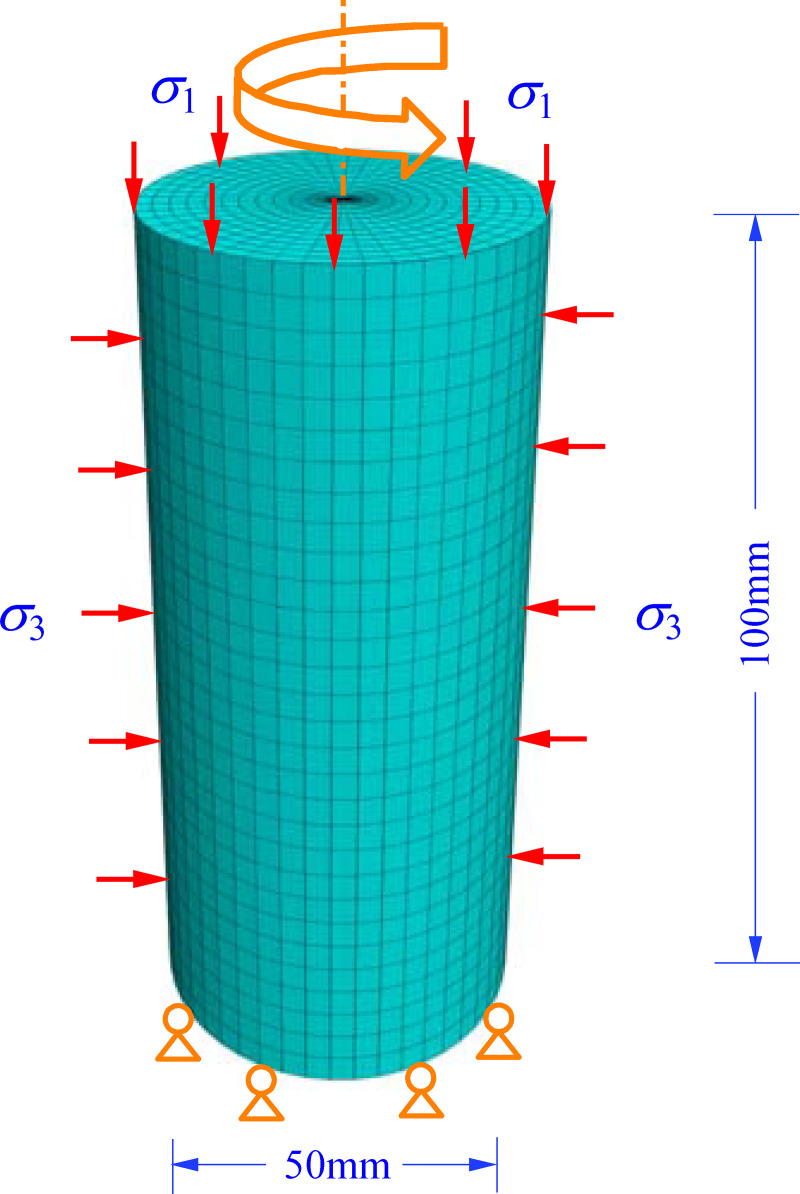
Numerical model and boundary conditions.

Using FLAC^3D^ software, the nonlinear creep and strain softening coupling model (NBSS) is used to calculate the creep curve of mudstone. The numerical model and boundary conditions are shown in Fig 9. We use a 3D numerical specimen (50 mm in diameter and 100 mm in length) divided into 3100 elements. The bottom of the specimen is constrained and the upper part is loaded. The experimental uniaxial creep parameters are selected for calculation and the viscosity coefficients *η*_m_ and *η*_k_ adopt the fitted parameters of the nonlinear equation in Table 5. The nonlinear Burgers model (*η*_m_ (*σ*_3_, *η*), *η*_k_ (*σ*_3_)) is used to calculate the steady creep of mudstone. The nonlinear creep damage variable function *η*_D_ (*σ*_3_, *η*_m_, *η*) and constant *η*_D_ are used to calculate the accelerated creep of mudstone, yielding constant values of 0.061, 0.0635, 0.066 and 0.0685. FISH language is used in the calculation to obtain the nonlinear creep damage variable function *η*_D_ (*σ*_3_, *η*_m_, *η*).

### Model verification

**Fig 10:**
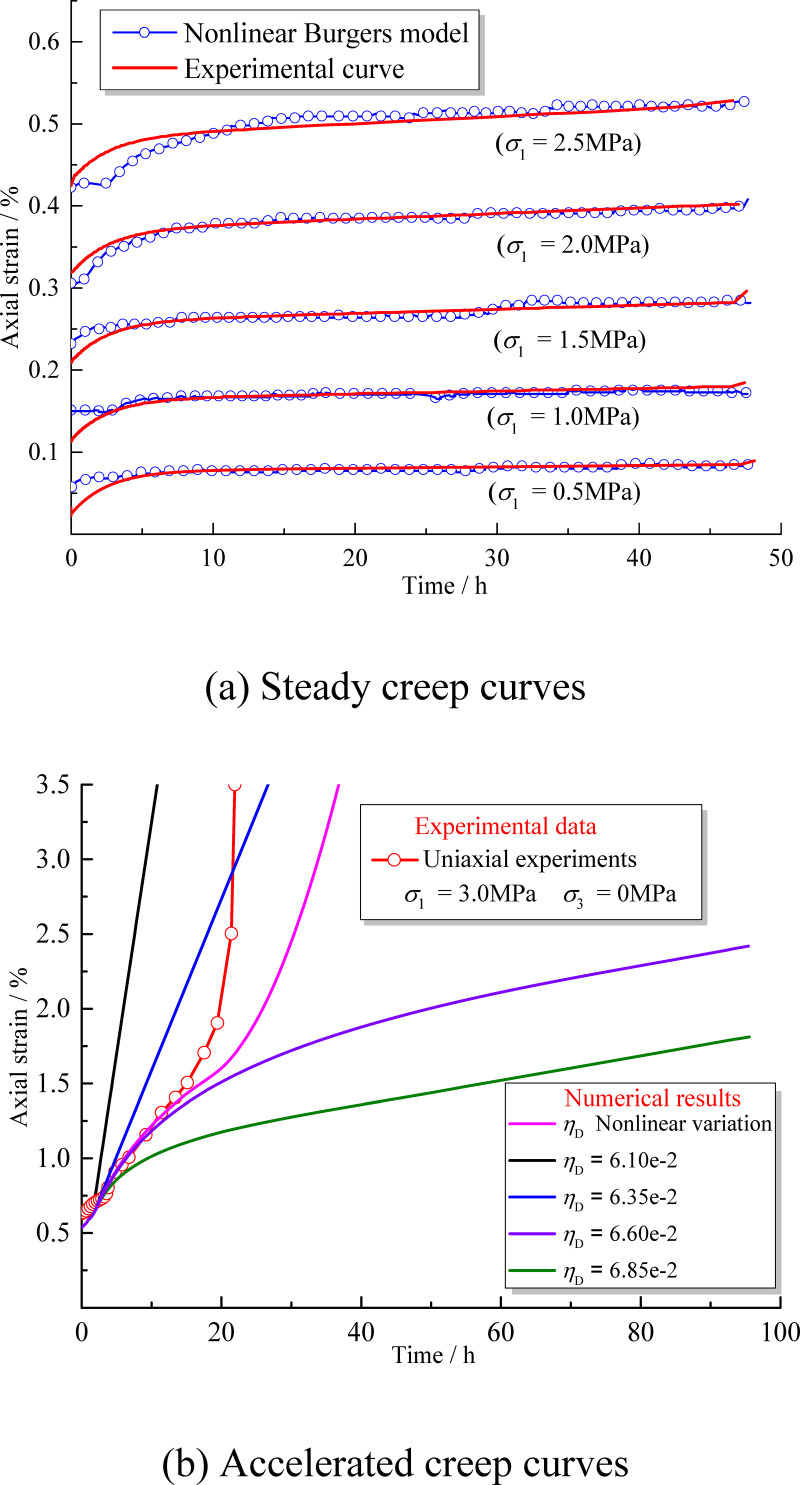
NBSS model curves alongside experimental curves. (a) Steady creep curves (b) Accelerated creep curves.

The results of NBSS numerical model were compared with the experimental data of yellow mudstone uniaxial creep to verify the reliability of the model. The steady creep curve of mudstone calculated by the NBSS numerical model is shown in Fig 10A. The numerical results are in good agreement with the experimental data in the decay creep and steady creep stages, which shows that the nonlinear Burgers model (*η*_m_ (*σ*_3_, *η*), *η*_k_ (*σ*_3_)) based on the variation of confining pressure is highly reliable. The accelerated creep curve is obtained by the calculation, as shown in Fig 10B. When the creep damage variable *η*_D_ is constant, the creep curves obtained by the numerical calculation show different characteristics. When *η*_D_ ≤ 0.0635, the creep curve obtained by the numerical calculation shows notable acceleration and linear characteristics. When *η*_D_ ≥ 0.066, the acceleration characteristic of the creep curve obtained by the numerical calculation is not apparent and tends to be in a steady stage. This shows that the accelerated creep characteristics of mudstone are highly sensitive to changes of *η*_D_. The creep curve calculated by the nonlinear function *η*_D_ (*σ*_3_, *η*_m_, *η*) shows clear acceleration and nonlinear characteristics. The creep acceleration start-up time of the numerical calculation is longer than that of the experimental data; however, the trends are very close, which demonstrates the high accuracy of the calculations. The NBSS numerical model can meet the accuracy requirements of the steady state and accelerated creep calculations for mudstone, and its nonlinear creep characteristics are more in line with actual project situations.

### Numerical simulation method

#### Numerical calculation method for coupled effect of creep and strain-softening

**Fig 11:**
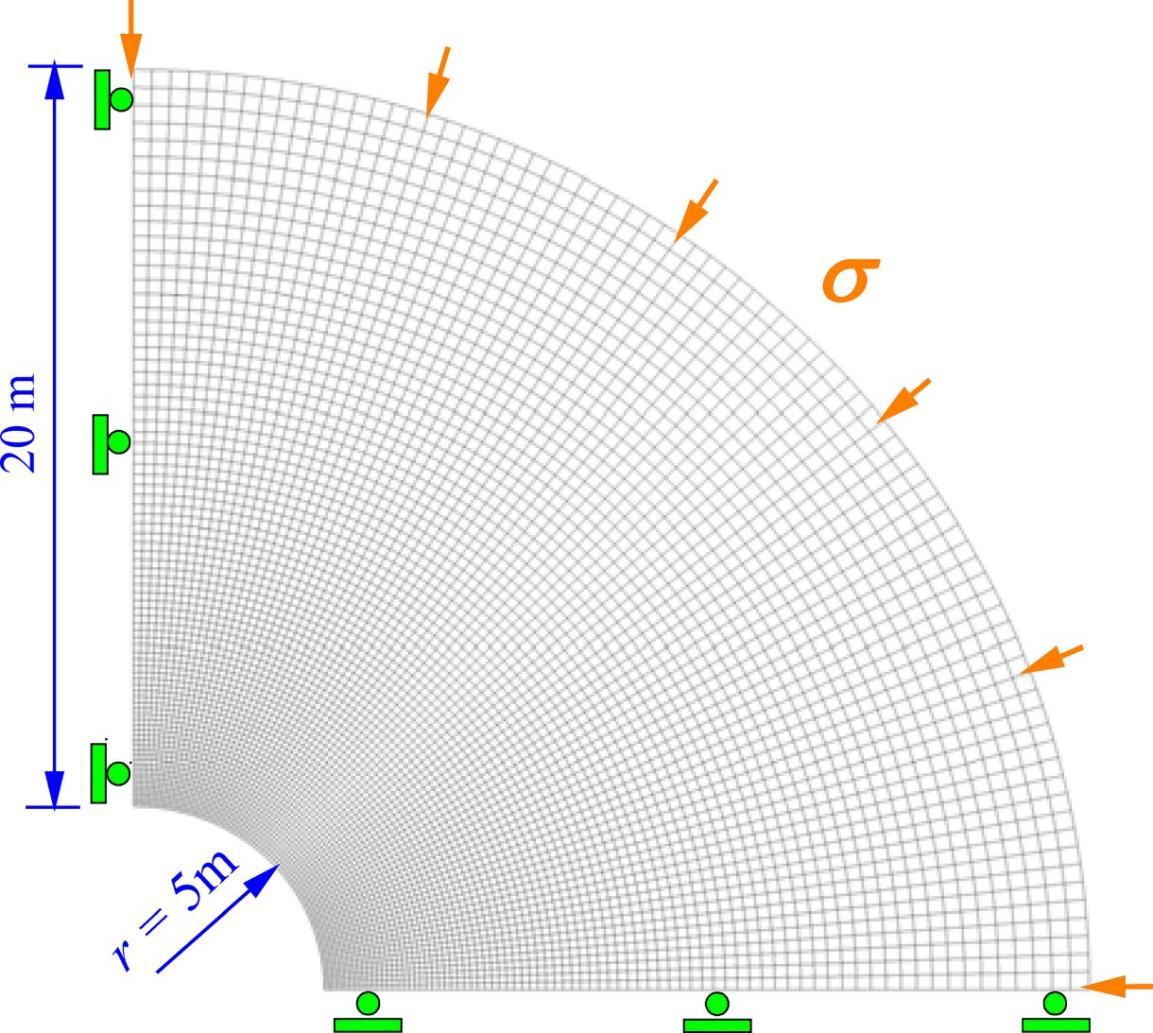
Dimensions and boundary conditions of the numerical model.

We constructed a typical numerical model of tunnel surrounding rock to analyze the coupled action law of creep and strain-softening. The size and boundary conditions of the numerical model are shown in Fig 11. The model is divided into 11,000 numerical elements and the rock surrounding the tunnel is in the state of hydrostatic pressure. The original rock stress is 2.5 MPa and the rock mechanical parameters are listed in Table 1. We apply two models in the calculation. The first is the NBMC, which does not take into account the strain-softening characteristics of mudstone after accelerated creep and the Burgers model parameters (*η*_m_, *η*_k_) have a nonlinear relationship with *σ*_3_. The second model is the NBSS, which takes into account the creep and strain-softening characteristics of the surrounding rock. The relationship between *η* and *σ*_3_ is nonlinear.

**Fig 12:**
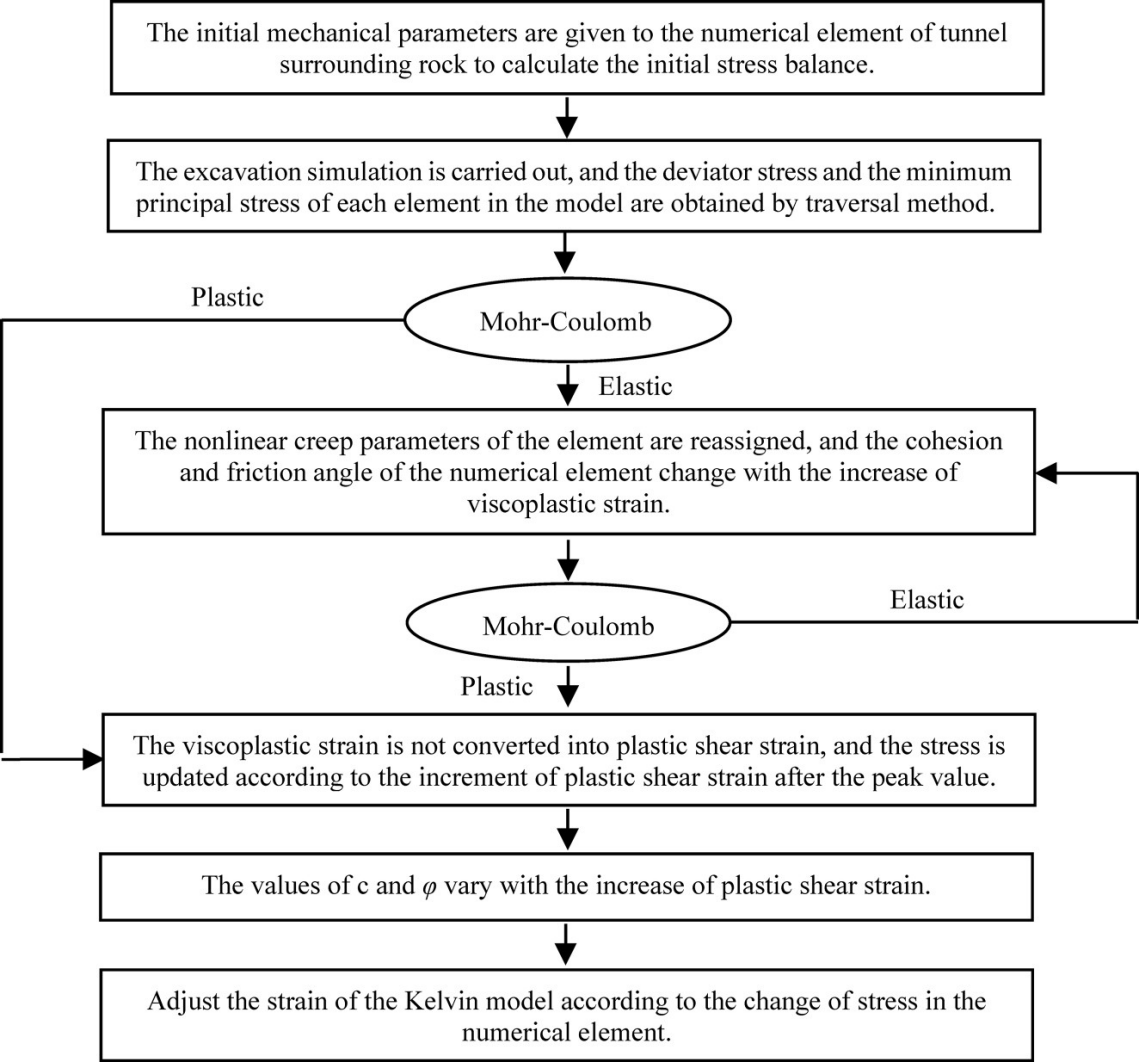
Numerical calculation flow chart of the coupled effect of creep and strain-softening. .

The numerical calculation process of the coupled effect of creep and strain-softening of rock surrounding a tunnel is as follows: First, the NBSS model and initial mechanical parameters are given to the numerical element to calculate the initial stress balance. An excavation simulation is then carried out and the deviator stress and minimum principal stress of each model element are obtained by the traversal method. The Mohr-Coulomb yield criterion is used to judge the elastic-plastic state of the numerical element. If the element is in an elastic state, the nonlinear creep parameters of the element are reassigned. The viscoplastic strain (*η*_m_ (*σ*_3_, *η*)) of the Maxwell component in the Burgers model is converted into plastic shear strain, and the *c* and *φ* values vary with increasing plastic shear strain. The Mohr-Coulomb yield criterion is then used to assess the elastic-plastic state of the numerical element. If the numerical element enters the plastic state, the viscoplastic strain is not converted into plastic shear strain. The stress is updated according to the plastic shear strain increment reaching after the peak value, and *c* and *φ* continue to vary with increasing plastic shear strain. In the numerical calculation, it is necessary to adjust the strain of the Kelvin model according to the stress change in the numerical element because the Kelvin model viscous strain does not vary when the stress field is adjusted. A flow chart of the coupled effect of creep and strain-softening calculation is shown in Fig 12.

### Rheological failure characteristics

#### Plastic zone distribution characteristics using the NBMC model

**Fig 13:**
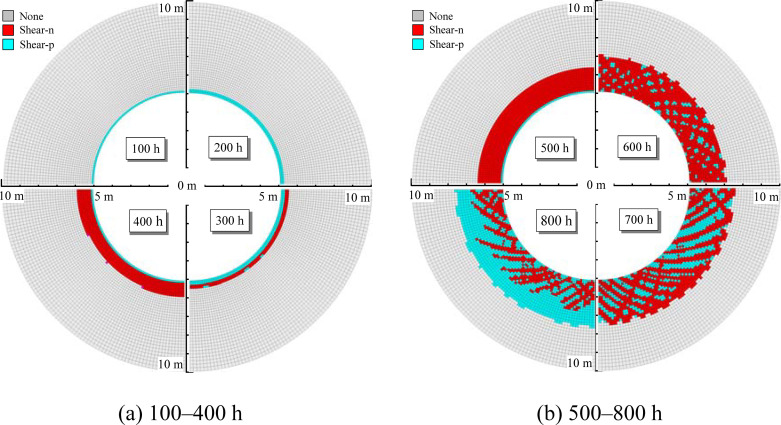
Evolution law of the plastic zone rock surrounding a tunnel (NBMC model). (a) 100–400 h (b) 500–800 h.

The nonlinear Burgers model is combined with Mohr-Coulomb model without considering the strain-softening characteristics of mudstone after accelerated creep. When the creep damage of the mudstone causes the surrounding rock to reach the Mohr-Coulomb yield strength, the surrounding rock will enter the plastic state. But the strength of the plastic surrounding rock will not attenuate with increasing plastic shear strain. The balance calculation is carried out after the excavation of the surrounding rock. The concentrated stress in the excavation boundary of the cavern does not exceed the mudstone strength. If the creep characteristics are not considered, there is no plastic zone in the surrounding rock. Under the conditions of no support, the evolution law of the plastic zone is calculated as shown in Fig 13. The plastic zone of the surrounding rock gradually expands over time. In the first 200 h, the plastic zone of the surrounding rock is small; between 300 and 500 h, the plastic zone notably increases obviously; and between 600 and 800 h, the expansion range of the plastic zone tends to gradually stabilize with a plastic zone thickness of 2.6 m.

#### Distribution characteristics of the plastic zone using the NBSS model

**Fig 14:**
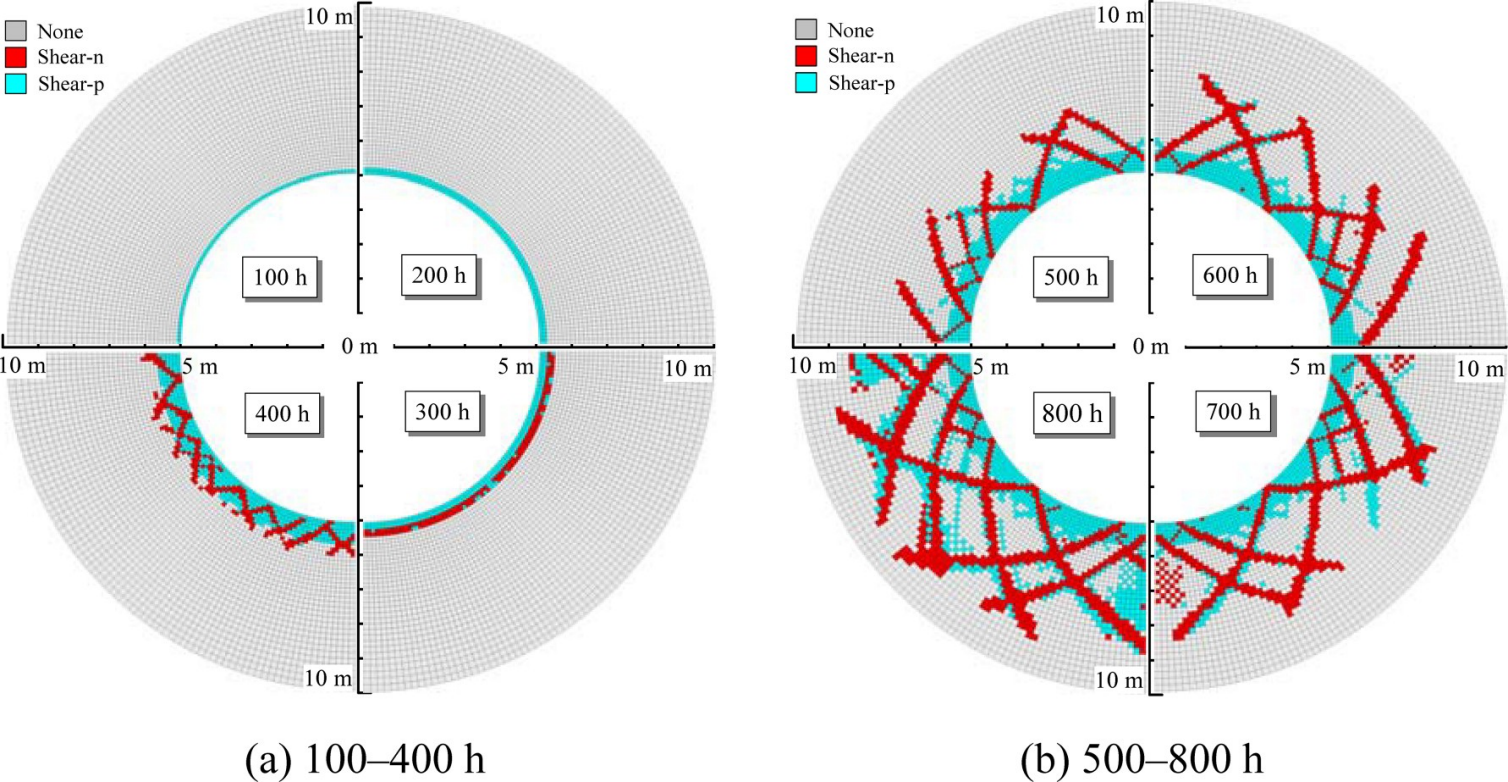
Evolution law of the plastic zone of rock surrounding a tunnel (NBSS model). (a) 100–400 h (b) 500–800 h.

The nonlinear Burgers model and strain-softening model are combined and consider the strain-softening characteristics of mudstone. When the creep damage causes the rock to reach the Mohr-Coulomb yield strength, the rock enters the plastic strain-softening state and the post-peak strength decreases with increasing plastic shear strain. The balance calculation is carried out after excavation. The concentrated stress in the excavation boundary of the cavern does not exceed the mudstone strength. If creep characteristics are not considered, there is no plastic zone in the surrounding rock. The evolution law of plastic zone of tunnel surrounding rock is calculated under the condition of no support, as shown in Fig 14. The plastic zone of the surrounding rock gradually expands with time. In the first 300 h, the range of the plastic zone is small, the maximum plastic zone thickness is 0.4 m, and the distribution is more uniform and complete. After 400 h, the plastic zone substantially increases and hosts the formation of localized fracture zones. When entering the strain-softening stage, the rock mechanical parameters in the local fracture zone strongly decrease, as shown in the red area in the Fig 14A. After 500 h, the plastic zone of the surrounding rock continues to increase. Localized fracturing is more apparent and the fracture zone gradually extends to the interior of the surrounding rock. After 700 h, the range of the plastic zone tends to gradually stabilize and the expanding thickness of the local fracture zone is 4.0 m.

**Fig 15:**
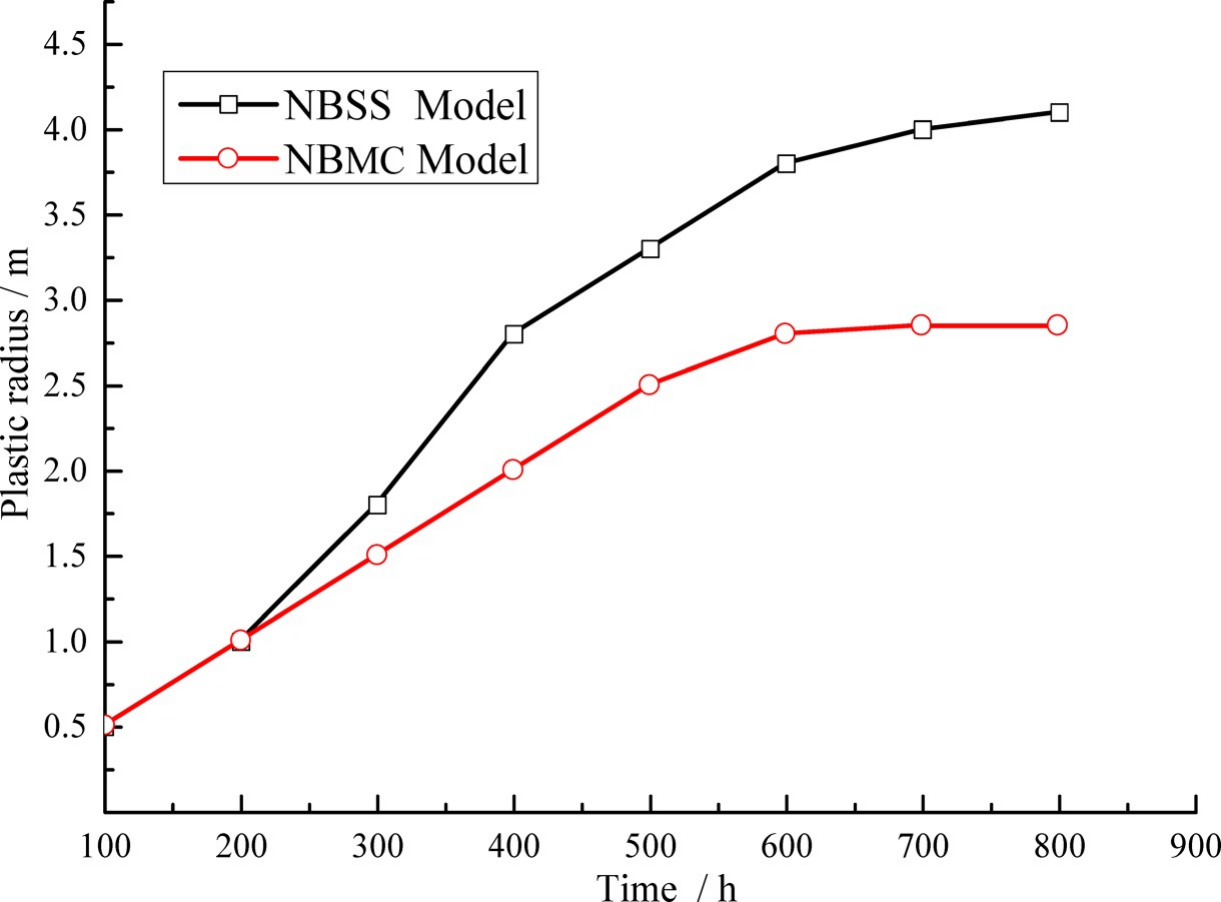
Variation of plastic zone thickness with time.

The above analysis shows that the plastic zone distribution characteristics obtained by the NBSS and NBMC models differ substantially. Fig 15 shows the variation of plastic zone thickness with time calculated by the two models. When the NBMC model is used to calculate the plastic zone, the distribution is relatively uniform and the development range is small, which is consistent with theoretical analysis. When the NBSS model is used, the creep damage causes the surrounding rock to enter the plastic state, the strength of the surrounding rock continues to decline with the plastic shear strain, and the plastic zone of the surrounding rock is larger with more local fracture characteristics.

**Fig 16:**
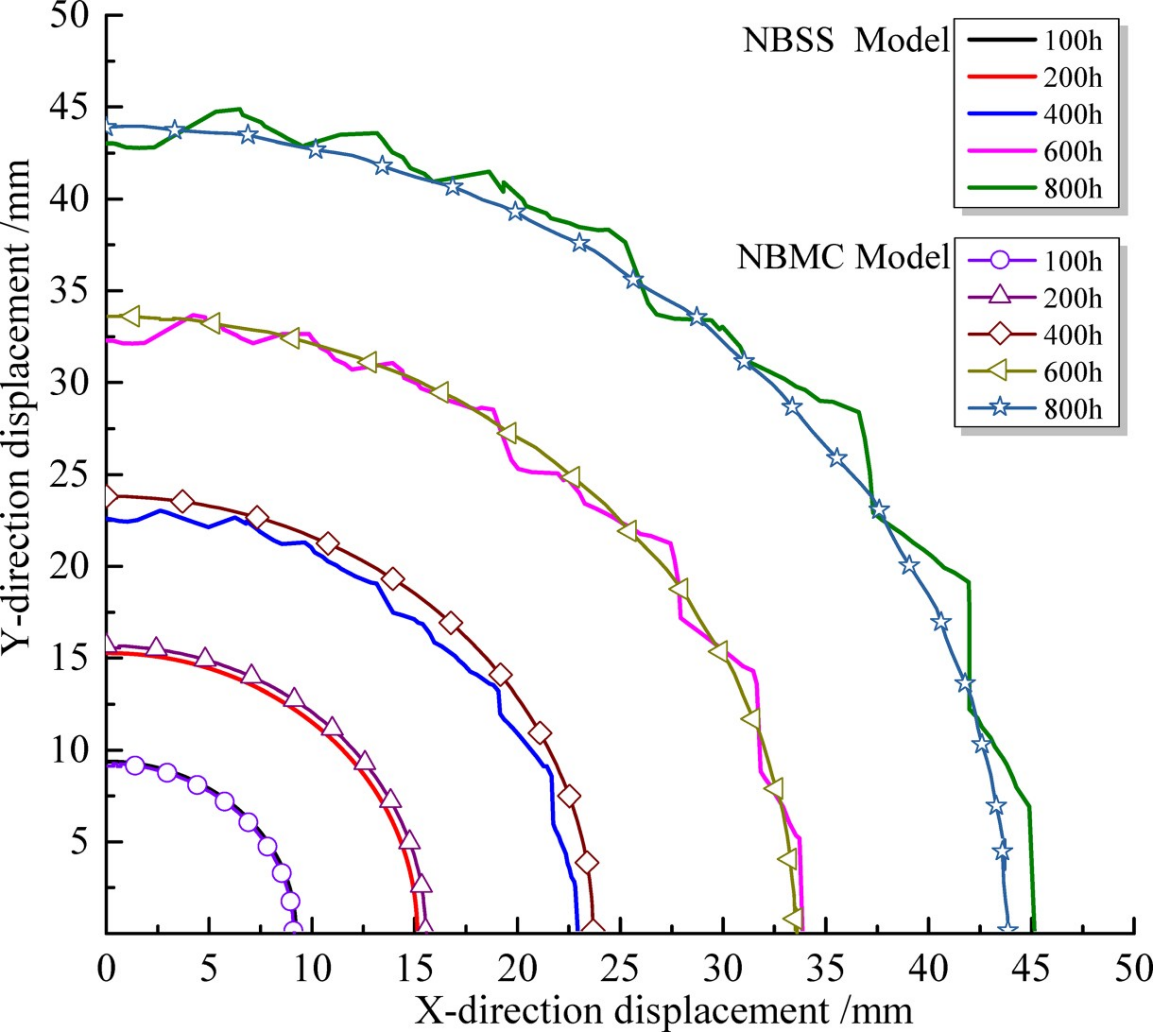
Deformation of rock surrounding a tunnel over time.

Fig 16 shows the variation of surrounding rock deformation with time under different model conditions. Within the first 100 h, the surrounding rock deformation of the two models is basically the same. With increasing time, the surrounding rock deformation obtained by the NBSS model is slightly smaller than that of the NBMC model over the range of 300–500 h. This is mainly because over this time range, the plastic zone distribution calculated by the NBSS model is discontinuous and the surrounding rock strength in the plastic zone remains in the strain-softening state. Between 600 and 800 h, the deformation of the surrounding rock obtained by the NBSS model gradually exceeds that of the NBMC model. The deformation of the surrounding rock at the cavern edge is discontinuous with clear nonlinear characteristics. The calculation results of the two models are similar when analyzing the rock deformation characteristics. The main difference lies in the failure characteristics of the plastic zone and deformation discontinuity. The reason is when the creep damage of mudstone makes the surrounding rock reach the Mohr-Coulomb yield strength, the surrounding rock of NBMC model enters the plastic state, however, the strength of the surrounding rock in the plastic state does not decrease with the increase of the plastic shear strain. The surrounding rock of NBSS model enters the plastic strain softening state, and the strength of surrounding rock decreases with the increase of plastic shear strain after the peak value.

#### Plastic zone distribution characteristics under anchor rod support conditions (NBSS)

**Fig 17:**
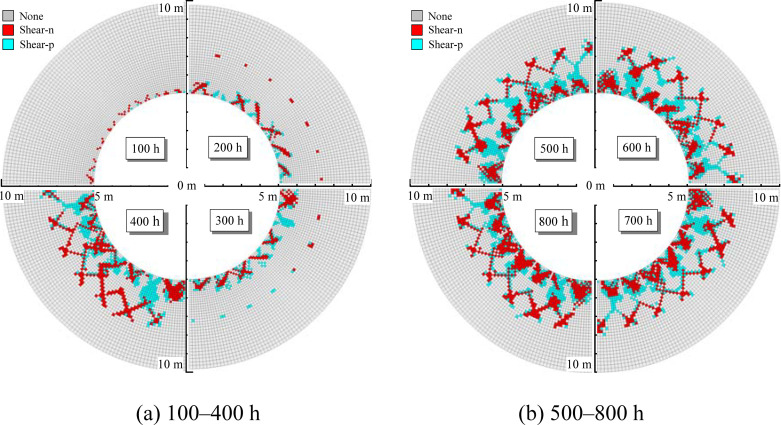
Plastic zone distribution characteristics of surrounding rock under anchor rod support. (a) 100–400 h (b) 500–800 h.

Anchor rods are used to support the surrounding rock and their stress characteristics and influence on the plastic zone are analyzed under these conditions using the NBSS model [39]. We adopted full-length bonded anchor rods with a diameter of 18 mm, length of 2.2 m, and spacing of 1.0 m as the support structure. The calculation result is shown in Fig 17. Under anchor rod support conditions, the expansion range of the plastic zone is well controlled. Within 300 h, the expansion range of the plastic zone is small; after 400 hours, the surrounding rock in the anchorage zone appears to locally fracture. However, with increasing time, the range of the plastic zone does not further expand. The anchor rods therefore play a superior supporting role and effectively control the development of plastic zone.

**Fig 18:**
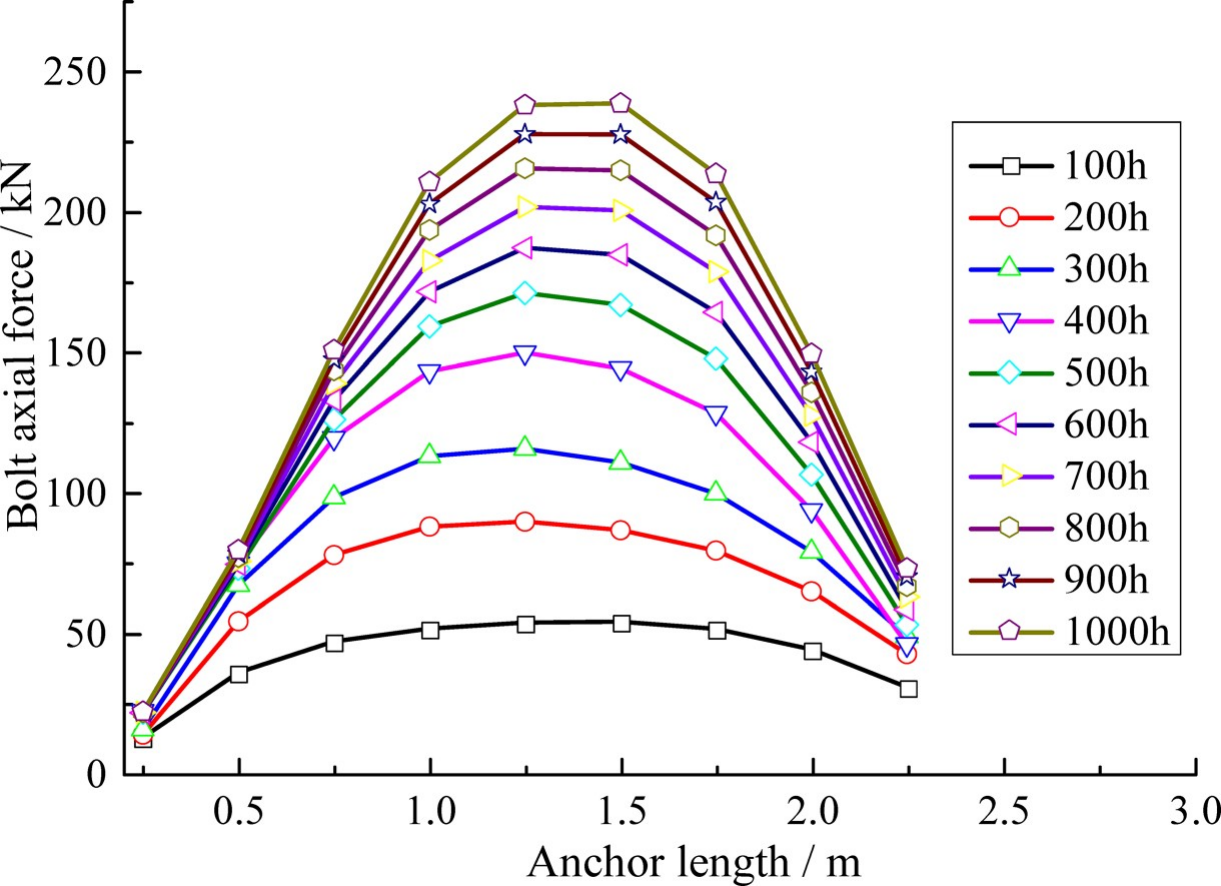
Anchor rod axial force as a function of time.

Fig 18 shows the evolution process of the anchor rod axial force with time. The anchor rod axial force is small in the first 300 h but gradually increases after 400 h even though the extension range of the plastic zone is effectively controlled by the anchor rod. After 500 h, the growth rate of the anchor rod axial force gradually decreases. The maximum anchor rod axial force is 240 kN at 1000 h, which is concentrated at an anchor rod length of approximately 1.5 m.

## Discussion

In order to study the deformation law of soft surrounding rock and provide a theoretical basis for soft rock tunnel support engineering, this paper firstly analyzes the creep characteristics of yellow mudstone through laboratory tests, and proposes a nonlinear creep and strain softening coupling model (NBSS). Then FLAC^3D^ finite difference software was used to numerically solve the NBSS model to verify the reliability of the model. Finally, using FLAC^3D^ finite difference software, NBMC model and NBSS model respectively to calculate the plastic zone distribution characteristics and deformation law of tunnel surrounding rock.

In rock creep damage research, many scholars’ research focuses on how to determine the creep damage variable, and put forward the expression of accelerated creep of rock mechanics model, but there is no in-depth study intensity attenuation laws, and in terms of the new model is put forward and the design is not enough comprehensive has limitations, their calculations often don’t meet a complex rheological properties of the soft rock in actual engineering. The nonlinear creep and strain softening coupling model (NBSS) proposed in this paper is different from other research results by avoiding the above research shortcomings and completing the research loopholes. (1) The research of soft rock creep and strain softening coupling rule. (2) The strength attenuation law of soft rock after accelerated creep is reflected in the numerical calculation. (3) The unique result is that the creep damage model of NBSS is more suitable for the analysis of the supporting system of weak surrounding rock in practical engineering because of its larger plastic zone and obvious local fracture characteristics.

Although the proposed NBSS model can reflect the coupled characteristics of rock mass creep and strain-softening, some limitations remain. For instance, the model is more suitable for underground tunnel engineering with relatively intact rock mass but not for jointed or broken rock. The NBSS model can be developed in further research based on discrete element software (e.g., UDEC or 3DEC). The strength damage characteristics of the structural plane can be reflected by constructing the model with a strength parameter that decays with time. The NBSS model can be used to reflect the creep and strain-softening characteristics of intact rock blocks and thus improves the applicability of the numerical method to practical engineering.

## Conclusions

We used theoretical analysis methods to reveal the coupled mechanism of creep and strain-softening of rock surrounding a tunnel. The strain-softening and creep damage characteristics of mudstone were obtained by laboratory experiments. We construct a nonlinear Burgers model and Mohr-Coulomb strain-softening model, and propose a nonlinear Burgers strain-softening model. The numerical calculation of the coupled effects of creep and strain-softening is detailed and the rheological failure characteristics of a circular tunnel surrounding rock are analyzed using FLAC^3D^. The following conclusions are obtained from the analysis.

1. The brittle-ductile characteristics of mudstone are apparent after reaching the peak value, and the confining pressure *σ*_3_ strongly influences the post-peak softening modulus *M*. The nonlinear exponential equation of *σ*_3_ and *M* obtained by fitting and nonlinear strain-softening mechanical model reflect the post-peak strength attenuation characteristics of mudstone and can also be used as pre-peak creep damage variable parameters. The fitted creep curves at different stress levels show high accuracy. The viscoplastic creep parameters (*η*_m_, *η*_k_) of mudstone are highly sensitive to *σ*_3_ and show notable nonlinear characteristics. We establish the nonlinear exponential equation of viscoplastic creep parameters and *σ*_3_ of mudstone. The proposed nonlinear creep damage model is more suitable for numerical development and calculation and more consistent with the evolution characteristics of the stress and displacement fields of complex rock environments in practical engineering.
2. The steady creep curve calculated by the nonlinear Burgers model is consistent with the experimental data, but this model does not reflect the accelerated creep characteristics of mudstone. By taking the softening coefficient *η* of mudstone as a creep damage variable, *η* is a constant when variable *σ*_3_ is not considered. *η* is a nonlinear time-varying function when considering variable *σ*_3_. Two kinds of creep damage variables are used to calculate the accelerated creep characteristics of mudstone. The accelerated creep curve calculated using the nonlinear creep damage variable shows good agreement with the experimental data. When a constant creep damage variable is used, the accelerated creep characteristics can be obtained but the acceleration curve shows linear characteristics. Therefore, the nonlinear creep and strain softening coupling model (NBSS) of mudstone is more suitable for analyzing the rheological law of surrounding rock in actual engineering.
3. The nonlinear Burgers Mohr-Coulomb model (NBMC) and nonlinear Burgers strain-softening (NBSS) model are used to calculate the plastic zone and deformation law of rock surrounding a tunnel. There are substantial differences between the two models in the distribution characteristics of the plastic zone. When using the NBMC creep damage model, the distribution of plastic zone is uniform and the plastic zone range is small; whereas the plastic zone calculated by the NBSS creep damage model is larger with notable localized fracture characteristics. The extension range of the plastic zone can be effectively controlled by anchor rod supports, but the axial force of the anchor rod increases with time. Anchor rod support does not meet the stability requirements of surrounding rock, thus it is necessary to combine supports to control the rheological deformation of surrounding rock.

The study in this paper is more suitable for analyzing the rheological law of complex and weak surrounding rock in practical engineering and provides a theoretical analysis basis for tunnel support of complex and weak surrounding rock.

## Supporting information

S1 File Experimental process and results. (DOCX)

## Abbreviations

*ω^p^*: The peak strength parameter
*ω^r^*: The residual strength parameter
*Y*: The target parameter
*σ_3_*: The confining pressure
a, b, and c: The constants obtained by the fitting
*J*: The creep compliance
*t*: The creep time
*i*: The number of hierarchical loading stages
*y*: The creep strain
*σ*_c_: The uniaxial compressive strength of the rock
$\varepsilon_{1}^{p}$: The maximum plastic strains
$\varepsilon_{3}^{p}$: The minimum plastic strains
$\varepsilon_{1}^{p,e}$: The maximum elastic principal strain prior to reaching the peak value
$\varepsilon_{1}^{drop}$: The softening strain after reaching the peak value
$\varepsilon_{1}^{e}$: The maximum elastic principal strain
$\sigma_{1}^{p}$: The peak principal stress
$\sigma_{1}^{r}$: The residual principal stress
${\varepsilon \&}_{j}^{p}$: The plastic principal strain rate
*σ_t_*: The tensile strength
*η**: The then simultaneously obtained
$\Delta \varepsilon_{j}^{ps}$: The main plastic shear strain increment

## References

[1] Hoek E, and, et al. Practical estimates of rock mass strength[J]. International Journal of Rock Mechanics & Mining Sciences, 1997. doi: 10.1016/s1365-1609(97)80069-x

[2] Oreste P Distinct analysis of fully grouted bolts around a circular tunnel considering the congruence of displacements between the bar and the rock[J]. International Journal of Rock Mechanics & Mining Sciences, 2008, 45(7):1052–1067. doi: 10.1016/j.ijrmms.2007.11.003

[3] Andargoli M E, Shahriar K, Ramezanzadeh A, et al. The analysis of dates obtained from long-term creep tests to determine creep coefficients of rock salt[J]. Bulletin of Engineering Geology & the Environment, 2018. doi: 10.1016/j.ijmst.2015.11.014 27648341 PMC5020562

[4] Alejano L.R., Rodriguez-Dono A., Alonso E., Manin G.F. Ground reaction curves for tunnels excavated in different quality rock masses showing several types of postfailure behaviour. Tunn. Undergr. Space Technol., 2009, 24 (6), 689–705. doi: 10.1016/j.tust.2009.07.004

[5] Yu J., Yao W., Duan K., Liu X.Y., Zhu Y.L. Experimental study and discrete element method modeling of compression and permeability behaviors of weakly anisotropic sandstones. Int. J. Rock Mech. Min. Sci, 2020 134, 104437. 10.1016/j.ijrmms.2020.104437.

[6] Alejano L.R., Alonso E., Rodriguez-Dono A., Fernandez-Manin G. Application of the convergence-confinement method to tunnels in rock masses exhibiting Hoek-Brown strain-softening behaviour. Int. J. Rock Mech. Min. Sci, 2010, 1 (47), 150–160. doi: 10.1016/j.ijrmms.2009.07.008

[7] Tomanovic Z. Rheological model of soft rock creep based on the tests on marl. Mech. Time-Dependent Mater, 2006, 10 (2), 135–154. doi: 10.1007/s11043-006-9005-2

[8] Song F., Wang H.N., Jiang M.J. Analytical solutions for lined circular tunnels in viscoelastic rock considering various interface conditions. Appl. Math. Model, 2018, 55,109–130. doi: 10.1016/j.apm.2017.10.031

[9] Barla G., Debernardi D., Sterpi D. Time-dependent modeling of tunnels in squeezing conditions. Int J Geomech., 2012, 12, 697–710. doi: 10.1061/(asce)gm.1943-5622.0000163.

[10] Mishra B., Verma P. Uniaxial and triaxial single and multistage creep tests on coal-measure shale rocks. Int J Coal Geol, 2015, 137, 55–65. doi: 10.1016/j.coal.2014.11.005

[11] Zhang Q., Wang H.Y., Jiang Y.J., Lu M.M., Jiang B.S. A numerical large strain solution for circular tunnels excavated in strain-softening rock masses. Comput. Geotech., 2019, 114, 103142. doi: 10.1016/j.compgeo.2019.103142

[12] Xu H.Y., Jiang X.Y. Creep constitutive models for viscoelastic materials based on fractional derivatives. Comput. Math. with Appl., 2017, 73(6), 1377–1384. doi: 10.1016/j.camwa.2016.05.002

[13] Markelova A., Trifonov A., Olkhovskaya V. Two-phase Filtration model for nonlinear viscoplastic oil and hard water drive. Appl. Mech. Mater, 2014, 698, 679–682. doi: 10.4028/www.scientific.net/amm.698.679

[14] Zhou H.W., Wang C.P., Han B.B., Duan Z.Q. A creep constitutive model for salt rock based on fractional derivatives. Int. J. Rock Mech. Min. Sci., 2011, 48(1), 116–121. doi: 10.1016/j.ijrmms.2010.11.004

[15] Di Mino G., Airey G., Di Paola M. Pinnola F.P., D’Angelo G., Lo Presti D. Linear and nonlinear fractional hereditary constitutive laws of asphalt mixtures. J. Civ. Eng. Manag, 2016, 22(7), 882–889. doi: 10.3846/13923730.2014.914104.

[16] Su T., Zhou H.W., Zhao J.W. A creep model of rock based on variable order fractional derivative. Chin. J. Rock Mech. Eng, 2019, 38(07), 1355–1363. doi: 10.13722/j.cnki.jrme.2018.1382.

[17] Wang M.Z., Cai M. A grain-based time-to-failure creep model for brittle rocks. Comput. Geotech, 2020, 119, 103344. doi: 10.1016/j.compgeo.2019.103344

[18] Wang H.N., Nie G.H. Analytical expressions for stress and displacement fields in viscoelastic axisymmetric plane problem involving time-dependent boundary regions. Acta Mech, 2010, 210 (3–4), 315–330. doi: 10.1007/s00707-009-0208-x

[19] Xu G.W., He C., Yan J., Ma G.Y. A new transversely isotropic nonlinear creep model for layered phyllite and its application. B. Eng. Geol Environ, 2019, 78 (7), 5387–5408. doi: 10.1007/s10064-019-01462-w

[20] Gioda G., Sterpi D. Visco-plastic behaviour around advancing tunnels in squeezing rock. Rock Mech. Rock Eng, 2009, 42(2), 319–339. doi: 10.1007/s00603-007-0137-8.

[21] Zhao Y.L., Wang Y.X., Wang W.J., Wan W., Tang J.Z. Modeling of non-linear rheological behavior of hard rock using triaxial rheological experiment. Int. J. Rock Mech. Min. Sci, 2017, 93, 66–75. doi: 10.1016/j.ijrmms.2017.01.004

[22] Zhao Y.L., Zhang L.Y., Wang W.J., Wan W., Li S.Q., Ma W.H., et al. Creep behavior of intact and cracked limestone under multi-level loading and unloading cycles. Rock Mech. Rock Eng, 2017, 50(6), 1409–1424. doi: 10.1007/s00603-017-1187-1

[23] Fu T.F., Xu T., Heap M.J., Meredith P.G., Mitchell T.M. Mesoscopic time-dependent behavior of rocks based on three-dimensional discrete element grain-based model. Comput. Geotech, 2020, 121, 103472. doi: 10.1016/j.compgeo.2020.103472

[24] Manica M., Gens A., Vaunat J., Ruiz D.F. A time-dependent anisotropic model for argillaceous rocks. Application to an underground excavation in Callovo-Oxfordian claystone. Comput. Geotech, 2017, 85, 341–350. doi: 10.1016/j.compgeo.2016.11.004

[25] Zhang J.F., Li D., Wang Y.H. Predicting tunnel squeezing using a hybrid classifier ensemble with incomplete data. B. Eng. Geol Environ, 2020, 79 (6), 3245–3256. doi: 10.1007/s10064-020-01747-5

[26] Alejano L.R., Rodriguez-Dono A., Veiga M. Plastic radii and longitudinal deformation profiles of tunnels excavated in strain-softening rock masses. Tunn. Undergr. Space Technol, 2012, 30, 169–182. doi: 10.1016/j.tust.2012.02.017

[27] Alonso E., Alejano L.R., Varas F., Fdez-Manin G., Carranza-Torres C. Ground response curves for rock masses exhibiting strain-softening behaviour. Int. J. Numer. Anal. Meth. Geomech, 2003, 27 (13), 1153–1185. doi: 10.1002/nag.315

[28] Krajcinovic D., Silva M.A.G. Statistical aspects of the continuous damage theory. Int. J. Solids Struct, 1982, 18(7), 551–562. doi: 10.1016/0020-7683(82)90039-7

[29] Krajcinovic D. Continuous damage mechanics. Applied Mechanics Review, 1984, 37(1), 1–6.

[30] Sun C., Ao Y.H., Wang L.G. The research on strain-softening characteristics and local fracture law of deep granite roadway. Complexity, 2020, 1064016. doi: 10.1155/2020/1064016

[31] Feng W.L., Qiao C.S., Wang T., Yu M.Y., Niu S.J., Jia Z.Q. Strain-softening composite damage model of rock under thermal environment. B. Eng. Geol Environ, 2020, 79 (8), 4321–4333. doi: 10.1007/s10064-020-01808-9.

[32] Wang S.L., Yin X.T., Tang H., Ge X.R. A new approach for analyzing circular tunnel in strain-softening rock masses. Int. J. Rock Mech. Min. Sci, 2010, 47 (1),170–178. doi: 10.1016/j.ijrmms.2009.02.011

[33] Balbaert I. & Joos P. The dynamic surface tension and the boltzmann superposition principle, 1989, (3). doi: 10.1016/0166-6622(89)80339-7

[34] Damjanac B., Fairhurst C. Evidence for a long-term strength threshold in crystalline rock. Rock Mech. Rock Eng, 2010, 43 (5), 513–531. doi: 10.1007/s00603-010-0090-9

[35] Pellet F., Hajdu A., Deleruyelle F., Besnus F. A viscoplastic model including anisotropic damage for the time dependent behaviour of rock. Int. J. Numer. Anal. Meth. Geomech, 2005, 29 (9), 941–970. doi: 10.1002/nag.450

[36] Wang H C, Zhao W H, Sun D S & Guo B B. Mohr-Coulomb yield criterion for rock plastic deformation. Chinese Journal of Geophysics (in Chinese), 2012, 4231–4238. doi: 10.6038/j.issn.0001-5733.2012.12.034.

[37] Zhao X.G., Cai M. A dilation angle model for rocks. Int. J. Rock Mech. Min. Sci, 2010, 47(3), 368–384. doi: 10.1016/j.ijrmms.2009.12.007

[38] Zhao Y.L., Tang J.Z., Fu C.C., Wan W., Luo S.L. Rheological test of separation between viscoelastic-plastic strains and creep damage model. Chin. J. Rock Mech. Eng, 2016, 35(7), w1297–1308. doi: 10.13722/j.cnki.jrme.2015.1178

[39] Ghosh Nilabjendu, Agrawal Harshit, Satyendra Kumar Singh & Gautam Banerjee. Optimum Chain Pillar Design at the Deepest Multi-Seam Longwall Workings in India. Mining, Metallurgy & Exploration: An Official International Peer-reviewed Journal of the Society, 2020, (5). doi: 10.1007/s42461-019-00138-z

