# Supplementary material for: Analysis and numerical calculation of a coupled creep and strain-softening model for soft rock tunnels
Source: PLoS One. 2021 Aug 26;16(8):e0256243. doi: 10.1371/journal.pone.0256243 (PMC8389497; doi:10.1371/journal.pone.0256243)
Supplement: S1 File — (DOCX) [file pone.0256243.s001.docx]

Experimental process and results

Experimental study on creep and strain softening properties of mudstone

**Rock specimen**

Specimens were collected from the surrounding rock face of the DK77+684 section of the Milin tunnel on the Sichuan-Tibet railway. The content of clay minerals is more than 50%, the content of carbonate minerals is less than 25%, and the content of felsic minerals is less than 25%. The yellow mudstone is relatively dense, with an average porosity of 3.29% and water content of 15.7%. The surrounding rock was mainly yellow mudstone with distinct rheological properties. To study the deformation mechanism, we performed conventional triaxial compression tests to determine the rock strain-softening mechanical properties and triaxial creep tests to investigate the rock creep characteristics.

**Triaxial compression tests**

**Experimental protocol and results**

A British GDS high precision soft rock rheometer was used in the test, which included a computer and full servo motor control that automatically collected data. The system has good dynamic response function, including a 250-kN motor driven digital load rack, 32-MPa pressure/volume control system, local strain sensor, and multi-function test module. The rheometer has high testing accuracy and meets the requirements of conventional triaxial and creep tests. The experimental loading device and control system are shown in Fig. 1a, and the characteristics of the rock specimens after loading failure are shown in Fig. 1b.

(a) Experimental loading device and control system

(b) Rock specimen characteristics after failure

**Fig. 1** Experimental system and rock specimen characteristics after failure

The displacement control method was used in the triaxial compression tests and the confining pressures were set to 0, 1, 3, 7, and 10 MPa. The full stress-strain curves of the specimens under different confining pressures were obtained by applying an axial load at the loading rate of 0.1 MPa/min, as shown in Fig. 2. The elastic modulus of the mudstone shows good consistency under different confining pressures, varying between 0.86 and 0.92 GPa. Under uniaxial conditions, the specimens exhibited brittle failure characteristics and the post-peak strength approached zero. With increasing confining pressure, the stress-strain curve gradually flatted after the peak value. Under a confining pressure of 7 MPa, the samples show an ideal elastic-plastic state; at 10 MPa, the samples show strain hardening characteristics. The triaxial compression test results show a strong correlation between mudstone strength and confining pressure. The samples show clear brittle-ductile characteristics after reaching the peak value.

**Fig. 2** Full stress-strain curves of the mudstone

**Peak strength and residual strength parameters**

The mudstone strength curve based on the Mohr-Coulomb criterion is obtained by linear fitting the stress combination of peak strength and residual strength of mudstone (Hoek and Brown 1997), as shown in Fig. 3. The variance values, *R*^2^, of the fitted parameters are all above 0.95, which indicates high fitting accuracy. The peak and residual compressive strength, shear strength and tensile strength parameters based on Mohr-Coulomb criterion were calculated, as shown in Table 1.

**Fig. 3** Fitted curves of mudstone strength and softening modulus

**Table 1** Mudstone strength parameters

| Strength criterion | Mohr-Coulomb criterion | | | | |
| --- | --- | --- | --- | --- | --- |
| Strength parameters | *σ*_c_/MPa | *c*/MPa | *Φ*/^o^ | *R*^2^ | *σ*_t_/MPa |
| Peak | 4.6 | 1.62 | 16.5 | 0.993 | 0.36 |
| Residual | 0.5 | 0.45 | 18.2 | 0.991 |

We use *M* to represent the post-peak stress-strain of the mudstone, i.e. post-peak softening modulus, as shown in Fig. 2. The experimental analysis shows a nonlinear correlation between the post-peak softening modulus and confining pressure, which is obtained by nonlinear exponential fitting. The fitted equation is as follows:

$Y\text{=}a\text{e}^{(-\sigma_{3}/b)}+c$ (1)

where *Y* is the target parameter, which represents softening modulus *M* in this equation, $\sigma_{3}$is confining pressure, and *a*, *b*, and *c* are the constants obtained by the fitting (Table 2).

**Table 2** Fitted softening modulus of mudstone

| target parameter | Fitted parameters | | |  |
| --- | --- | --- | --- | --- |
| *Y* | *a* | *b* | *c* | *R*^2^ |
| *M* | 0.289 | 1.454 | 0.00681 | 0.985 |

**Creep tests**

**Experimental protocol**

Mudstone creep tests were carried out using a British GDS high precision soft rock rheometer. We adopted hierarchical loading and applied a maximum load of 80%–90% of the peak strength from the conventional triaxial compression tests under the same confining pressure. The tests were divided into 6–8 loading gradients, as shown in Table 3. During the creep tests, the confining pressure was loaded to the target value using a stress loading rate is 0.05 MPa/s and then held fixed. The axial compression was loaded to the target value of the first stage creep and held constant. The following load was applied every 48 h until the specimen failed.

**Table 3** Stress loading gradients under different confining pressure

| Confining pressure *σ*_3_/MPa | 0 | 1.0 | 3.0 | 7.0 |
| --- | --- | --- | --- | --- |
| Deviator stress (*σ*_1_-*σ*_3_)/MPa | 0.5 | 0.5 | 1.0 | 2.0 |

**Creep curve characteristics and fitted parameters**

Fig. 4 shows the axial creep curves of the mudstone under different confining pressures. The creep curve exhibits the typical three-stage creep characteristics of rock. When $\sigma_{3}$= 0, the mudstone shows characteristics of decay creep and steady creep while loading from stages 1 to 5. When the deviatoric stress reached 3 MPa, the mudstone entered the accelerated creep stage and the rock specimen was destroyed. The mudstone creep curves characteristics under different confining pressures show that the steady creep curve under high deviatoric stress gradually tends to flatten with the increasing confining pressure. This shows that confining pressure exerts a strong influence on the viscoplastic rheological properties of mudstone.

(a) σ_3_ = 0 MPa

(b) σ_3_ = 1 MPa

(c) σ_3_ = 3 MPa

(d) σ_3_ = 7 MPa

**Fig. 4** Creep curves and fitted curves of mudstone under different confining pressures

The Burgers model was used to fit and analyze the creep experimental curve of the mudstone. The Burgers model is composed of a Maxwell model and Kelvin model in series, which well reflects the decay creep and steady creep stage of rock. The specific methods are as follows: The GDS file obtained from the experiment was imported into an Excel file and the experimental data were processed by the Boltzmann superposition principle. The expression is as follows (Balbaert. 1989):

$\varepsilon_{T}(t)=\sum\varepsilon_{i}(t)=\sum\Delta\sigma_{i}J(t-t_{i})$ $t>t_{i}$ (2)

where *J* is the creep compliance, *t* is the creep time, and *i* is the number of hierarchical loading stages, which are taken as 1, 2, ..., *i* − 1.

The processed data were then imported into Origin software. Because the tests were performed in stages, the data were fitted step by step. The obtained nonlinear fitted equation of the Burgers model suitable for the hierarchical loading mode is given as:

$\begin{matrix} y=At+\sum[A(t-48(i-1))]+2B+ \\ C\left\{2-\exp(-Dt)-\sum[-D(t-48(i-1))]\right\} \end{matrix}$ (3)

where *t* is creep time, *y* is creep strain, *A* = *σ*/*η*_m_, *B* = *σ*/*E*_m_, *C* = *σ*/*E*_k_, and *D* = *E*_k_/*η*_k_. The model parameters *η*_m_, *η*_k_, *E*_m_, and *E*_k_ were inversely calculated according to the fitted results. The obtained parameters are listed in Table 4 and the fitted and experimental curves are compared in Fig. 3.

**Table 4** Fitted creep parameters of mudstone

| *σ*_3_/MPa | *η*_m_/GPa·h^-1^ | *η*_k_/GPa·h^-1^ | *E*_m_/GPa | *E*_k_/GPa |
| --- | --- | --- | --- | --- |
| 0 | 99.87 | 0.51 | 0.35 | 0.42 |
| 1 | 109.5 | 0.41 | 0.36 | 0.44 |
| 3 | 141.2 | 0.30 | 0.35 | 0.42 |
| 7 | 159.3 | 0.29 | 0.34 | 0.40 |

**Fig. 5** Viscosity coefficient of mudstone as a function of confining pressure

The effect of confining pressure on the viscosity coefficients based on the Maxwell model and Kelvin model is shown in Fig. 5. The viscosity coefficient $\eta_{k}$ decreases strongly with increasing confining pressure in the low pressure range (0–3 MPa) and tends to stabilize in the higher pressure range (3–7 MPa). The viscosity coefficient $\eta_{m}$ increases gradually with the increase of confining pressure. The relationship between the viscosity coefficient and confining pressure is obtained by exponential fitting analysis of the viscosity coefficients $\eta_{m}$ and $\eta_{k}$ from the Maxwell and Kelvin models, respectively. The data are fitted following Eq. (3) and the obtained parameters are listed in Table 5. The *R*^2^ of the fitted equations all exceed 0.95, which indicates high accuracy.

**Table 5** Fitted parameters of viscosity coefficient of mudstone

| Target parameters | Fitted parameters | | |  |
| --- | --- | --- | --- | --- |
| *Y* | *a* | *b* | *c* | *R*^2^ |
| *η*_k_ | 0.23 | 1.51 | 0.28 | 0.97 |
| *η*_m_ | -75.54 | 3.95 | 173.07 | 0.95 |
